# Differential processing of RNA polymerase II at DNA damage correlates with transcription-coupled repair syndrome severity

**DOI:** 10.1093/nar/gkae618

**Published:** 2024-07-18

**Authors:** Camila Gonzalo-Hansen, Barbara Steurer, Roel C Janssens, Di Zhou, Marjolein van Sluis, Hannes Lans, Jurgen A Marteijn

**Affiliations:** Department of Molecular Genetics, Oncode Institute, Erasmus MC Cancer Institute, Erasmus University Medical Center, Rotterdam, The Netherlands; Department of Molecular Genetics, Oncode Institute, Erasmus MC Cancer Institute, Erasmus University Medical Center, Rotterdam, The Netherlands; Department of Molecular Genetics, Oncode Institute, Erasmus MC Cancer Institute, Erasmus University Medical Center, Rotterdam, The Netherlands; Department of Molecular Genetics, Oncode Institute, Erasmus MC Cancer Institute, Erasmus University Medical Center, Rotterdam, The Netherlands; Department of Molecular Genetics, Oncode Institute, Erasmus MC Cancer Institute, Erasmus University Medical Center, Rotterdam, The Netherlands; Department of Molecular Genetics, Oncode Institute, Erasmus MC Cancer Institute, Erasmus University Medical Center, Rotterdam, The Netherlands; Department of Molecular Genetics, Oncode Institute, Erasmus MC Cancer Institute, Erasmus University Medical Center, Rotterdam, The Netherlands

## Abstract

DNA damage severely impedes gene transcription by RNA polymerase II (Pol II), causing cellular dysfunction. Transcription-Coupled Nucleotide Excision Repair (TC-NER) specifically removes such transcription-blocking damage. TC-NER initiation relies on the CSB, CSA and UVSSA proteins; loss of any results in complete TC-NER deficiency. Strikingly, UVSSA deficiency results in UV-Sensitive Syndrome (UV^S^S), with mild cutaneous symptoms, while loss of CSA or CSB activity results in the severe Cockayne Syndrome (CS), characterized by neurodegeneration and premature aging. Thus far the underlying mechanism for these contrasting phenotypes remains unclear. Live-cell imaging approaches reveal that in TC-NER proficient cells, lesion-stalled Pol II is swiftly resolved, while in CSA and CSB knockout (KO) cells, elongating Pol II remains damage-bound, likely obstructing other DNA transacting processes and shielding the damage from alternative repair pathways. In contrast, in UVSSA KO cells, Pol II is cleared from the damage via VCP-mediated proteasomal degradation which is fully dependent on the CRL4^CSA^ ubiquitin ligase activity. This Pol II degradation might provide access for alternative repair mechanisms, such as GG-NER, to remove the damage. Collectively, our data indicate that the inability to clear lesion-stalled Pol II from the chromatin, rather than TC-NER deficiency, causes the severe phenotypes observed in CS.

## Graphical Abstract

**Graphical Abstract:**
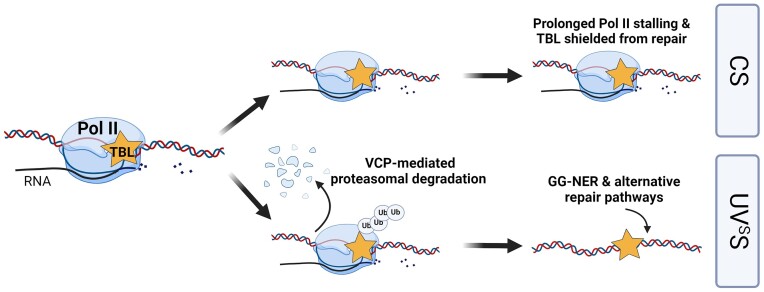

## Introduction

Unperturbed transcription of eukaryotic genes by RNA polymerase II (Pol II) is crucial for proper cell function. However, the DNA strand transcribed by Pol II is continuously threatened by agents of both endogenous and exogenous origins that cause damage that can severely impede or even completely block transcription (1–4). Endogenous transcription-blocking DNA damage can result from a variety of sources such as oxidative stress and aldehydes (5–7), but also transcription-replication conflicts or DNA-secondary structures may impede transcription (1,8). Well-known examples of exogenous DNA damaging agents that can induce transcription-blocking lesions (TBLs) are ultraviolet radiation (UV) and chemicals like cisplatin (3–5), which induce bulky DNA lesions that block the forward progression of elongating Pol II (3). Unresolved TBLs can lead to a transcription inhibition or transcription of aberrant RNA (9), causing cellular dysfunction, senescence and apoptosis, eventually resulting in damage-induced aging (2,10). To overcome these severe consequences, TBLs are efficiently removed by Transcription-Coupled Nucleotide Excision Repair (TC-NER), thereby safeguarding transcription (2,11,12).

Lesion-stalled Pol II is recognized by CSB (13), which uses its ATP-dependent translocase activity to discriminate between lesion-stalled Pol II and naturally-paused Pol II (14,15). The prolonged CSB-binding to Pol II is hypothesized to result in the recruitment of CSA (16). CSA is part of the Cullin 4-based CRL4^CSA^ ubiquitin E3-ligase, which upon activation by NEDD8 conjugation, ubiquitylates both CSB (17) and Pol II (18,19). Ubiquitylation of specifically the K1268 residue of RPB1 (18,20), the largest subunit of Pol II, which is stimulated by ELOF1 (21,22), drives efficient recruitment of the downstream TC-NER factors UVSSA and TFIIH (18,21–23). Due to its interaction with the de-ubiquitylating enzyme USP7, UVSSA inhibits CSB ubiquitylation thereby preventing its degradation (24,25). In addition, UVSSA recruits TFIIH by a direct interaction with the p62 subunit (16,26). Following its recruitment, TFIIH is bound by XPA, which stimulates the DNA damage verification activity of TFIIH (27), and the recruitment and orientation of the ERCC1/XPF and XPG nucleases to finally excise the TBL (28). After filling of the resulting single-stranded DNA gap by DNA polymerases, Pol II-mediated transcription can resume (2).

The biological relevance of TC-NER is best illustrated by the early onset of Cockayne syndrome (CS) in patients with inactivating mutations in the CSA and CSB genes (29). CS patients suffer from growth and development complications appearing within their first year of life, microcephaly and progressive neurodegeneration, photosensitivity, and symptoms of accelerated aging such as hearing loss, ophthalmological degeneration, osteoporosis, liver and kidney failure and motor abnormalities. Due to the severity of the disease, the average life expectancy of CS patients is only 12 years (30,31). Strikingly, the phenotypes observed in UV-sensitive syndrome (UV^S^S) patients, which have an equal TC-NER deficiency at the cellular level as CS patients, are remarkably milder (32,33). UV^S^S can be caused by mutations in UVSSA (24,25,34), but also by specific mutations in for example CSA (35). UV^S^S patients have mild cutaneous phenotypes including photo-hypersensitivity and freckling without the neurodegeneration and other severe phenotypes of CS (36). Thus far the underlying mechanism for this striking difference in phenotypes remains unclear, which may be due to the fact that most research is performed in cell lines derived from rare patients with different genetic backgrounds, complicating comparisons between these cells.

The observed phenotypical differences have been linked to additional functions of CS proteins compared to UVSSA. Several functions for CS proteins outside of TC-NER were described, mainly for CSB, including preservation of mitochondrial function (37,38), regulation of transcription mostly for neuronal development (39) and control of redox balance (40). However, the exact roles of CSA and UVSSA in these processes remain much less understood. Instead, the progressive nature of the CS phenotype is in line with the stochastic accumulation of endogenous DNA damage or toxic repair intermediates in TC-NER deficient cells, suggesting that a defective genome maintenance mechanism is the common denominator of this TC-NER linked disorder (2). An interesting observation is that deficiencies in the first initiation steps of TC-NER are characteristic of CS, due to mutations in CSA and CSB, while defective downstream steps are characteristic of UV^S^S, due to UVSSA mutations (24,25,34), or by mutations that affect the presence of UVSSA in the TC-NER complex (41,35). Mostly, in CS cells, the activity of the CRL4^CSA^ ubiquitin E3-ligase is impeded, for example due to reduced CSA binding caused by mutations at the CSB-CSA interface, or by mutations severely compromising CSA structure (15,29,42,43). As lesion-stalled Pol II is one of the key substrates of CRL4^CSA^ (18), TBL-bound Pol II will most likely not be ubiquitylated and subsequently degraded in CS cells. Indeed, it has been shown that Pol II degradation is severely reduced in CS cells (34,41). Therefore a model was hypothesized in which lesion-stalled Pol II will remain chromatin-bound in CS cells for a prolonged time as its degradation is hampered due to the impeded CRL4^CSA^ activity (2,34). Such chromatin-bound Pol II will not only create a toxic persistent transcription block, but will most likely also impede other DNA transacting processes, and will shield the TBL that is buried within Pol II (3) from alternative repair pathways. Likewise, this model also suggests that in UV^S^S cells, in which the CRL4^CSA^ E3 ubiquitin ligase complex remains active (35), lesion-stalled Pol II can still be degraded without repair of the lesion. This CRL4^CSA^-mediated degradation of lesion-stalled Pol II might eventually allow the access of alternative repair pathways to resolve the TBL. Therefore it is postulated that this differential processing of Pol II could explain the difference in phenotypes between CS and UV^S^S (2,12). However, thus far it remains to be clarified whether TBL-stalled Pol II fails to be degraded in CS cells and whether the CRL4^CSA^-mediated proteasomal degradation of Pol II is important for degradation of lesion-stalled Pol II in UV^S^S cells to allow access of alternative repair pathways to the TBL. Furthermore, whether and how Pol II is resolved form the chromatin upon deficiencies in more downstream TC-NER factors, like XPA, remains unknown.

To study the differential chromatin binding of Pol II at TBLs in TC-NER deficient cells, we generated knock-ins (KIs) of endogenously GFP-tagged Pol II (44) in isogenic knockout (KO) cells of the different TC-NER factors. By blocking *de novo* transcription initiation, this allowed us to study the residence time of chromatin-bound Pol II upon DNA damage using live-cell imaging approaches and cell fractionation assays (45). We show that in TC-NER proficient cells lesion-stalled Pol II is swiftly resolved by repair of the TBL. In line with our hypothesis, we find that in TC-NER deficient CSA and CSB KO cells lesion-stalled Pol II remains chromatin-bound, while in UVSSA KO cells Pol II is removed from the lesion by VCP-mediated proteasomal degradation. Together, our data proves that the prolonged stalling of Pol II at TBLs correlates with the CS phenotype, and suggests that the failure to remove toxic TC-NER intermediates will cause persistent transcription stress and that these intermediates shield TBLs from alternative repair pathways like GG-NER. As a consequence, prolonged stalling of Pol II at a TBL will contribute to cellular functional decline and increased apoptosis and senescence, which are the basis of the severe, progeroid phenotype of CS.

## Materials and methods

### Cell culture

MRC5 (sv40) fibroblasts and HCT116 colorectal cells were cultured in DMEM (Gibco) and Ham's F10 (Invitrogen) mixed 1:1 supplemented with 10% fetal calf serum (Biowest) and 1% Penicillin–Streptomycin at 37°C with 5% CO_2_.

MRC5 GFP-RPB1 knock-in (MRC5^GFP-RPB1^) TC-NER deficient cells were generated by targeting of the RPB1 locus as described in (44) in UVSSA KO (46), and CSA, CSB, XPA and XPC KOs, all described in (45). Sequences of used sgRNA for generating KO cells are indicated in Supplementary Table S1. Genomic DNA of single-cells clones of MRC5^GFP-RPB1^ TC-NER KO cells was isolated, the edited genomic locus was amplified by PCR (Supplementary Table S2), sequenced and frameshift mutations were verified by TIDE analysis (47). TC-NER KO was also verified with immunoblot.

MRC5^GFP-RPB1^ CSA/UVSSA double KO cells were generated by CRISPR/Cas9-mediated gene editing targeting UVSSA in CSA KO cells. KO of UVSSA was verified as discussed above. MRC5^GFP-RPB1^ UVSSA/XPC double KO cells were generated by transfecting UVSSA KO cells with a pLentiCRISPR.v2 plasmid containing sgRNA targeting XPC. Transfected cells were selected using 1 μg/ml Puromycin (Invitrogen) for 2 days. XPC KO was verified as discussed above. HCT116 knockout (KO) cells were generated by transfecting HCT116 osTIR1 CSB knock-in HCT116^CSB-mScarletI^ cells (21) with a pLentiCRISPR.v2 plasmid containing sgRNA targeting CSA or XPA (48) (Supplementary Table S1).

pEGFP-N1 CSA^WT^ and CSA mutant plasmids were generated as previously described (49). The ORFs were amplified with Q5 polymerase (Invitrogen) using primers (5′-TCCTTCTTCATCACTGCTGC-3′) and (5′-TCACTTGTCGTCATCGTCTTTGTAGTCTCCTTCTTCATCACTGCTGC-3′) adding a FLAG-tag at the C-terminus and cloned into pEntr-D-Topo (Invitrogen) according to the manufacturer's instructions. The CSA ORFs were transferred into a pLenti-CMV-puro vector (50) using Gateway® LR cloning kit (Invitrogen) and transfected in HEK293T for lentivirus production. The lentivirus was subsequently transduced in MRC5^GFP-RPB1^ CSA KO cells, generating CSA^WT^, CSA^A160T^, CSA^W194C^ and CSA^W361C^ complemented cells, which were selected with 5μg/ml puromycin.

DNA damage was introduced using the indicated doses of Illudin S (I019, TOKU-E) or by UV-C irradiation. UV-C-induced damage was generated by irradiation of cells with the indicated UV doses with UV-C light emitted by a 254 nm germicidal lamp (Philips) after washing with PBS.

The CDK7 inhibitor THZ1 (M60214-2s, Xcessbio) was used at a concentration of 2μM and added to cells 30 min post-damage for 45–90 min. Ribosome activity was inhibited with 100 μM cycloheximide (C4859_1ML, Sigma), added to cells 2 h prior to UV irradiation or Illudin S treatment and kept for the duration of the whole experiment. Proteasome and VCP inhibitors were added to cells at the time of UV-irradiation and kept on the cells for the duration of the experiment. Proteasome inhibitor MG132 (BML-PI102-0025, ENZO) was used at a concentration of 50 μM, Bortezomib (Velcade PS-341, F1200 UBPBio) at 5 μM and VCP inhibitor NMS-873 (S7285_5mg, Selleck Chemicals) at 5 μM.

For siRNA transfection, cells were transfected twice using 4 μL Lipofectamine RNAi Max (Invitrogen) and 4 μL siRNA (20 μM stock). Cells were transfected 2 days prior to harvesting/functional experiment according to the manufacturer's instructions. siRNAs were acquired from Horizon and sequences are listed in Supplementary Table S3. For transient overexpression of DDB2, cells were transfected with 2 μg DDB2-mCherry plasmid (51) using 5μL Lipofectamine 2000 Reagent (Invitrogen) according to manufacturer's instructions.

### Clonogenic survival assay

300–500 cells were seeded in 6-well plates in triplicate. The following day cells were mock- UV- or Illudin S-treated with the indicated doses and allowed to grow for 10–12 days. Illudin S was kept on the cells for 24 h, removed by a PBS wash step. Cells were fixed and stained with 50% methanol, 7% acetic acid, and 0.1% w/v Coomassie brilliant blue (Sigma). Colony numbers were determined using a GelCount colony scanner (Oxford Optronix). Average colony number for each UV dose was normalized to mock-treated conditions which were set to 1.

### Cell fractionation

Cells were seeded in 6 cm dishes, treated as desired and lysed for 30 min on ice in lysis buffer (30 mM HEPES pH 7.6, 1 mM MgCl_2_, 130 mM NaCl, 0.05% Triton X-100, 50 μM MG132, cOmplete EDTA-free protease inhibitor (Roche) and Phosphatase inhibitor cocktail II (Sigma-Aldrich). Cell lysates were fractioned by centrifugation at 15.000 g for 10 min and the supernatant (cytoplasmic fraction) was collected in a separate Eppendorf tube. The cell pellet (chromatin-bound fraction) was first quickly washed with lysis buffer followed by a subsequent prolonged wash step in lysis buffer for 10 min on ice. After a second centrifugation step (15.000 g 10 min) the pellet was resuspended in 75 μl lysis buffer. Next, chromatin was degraded with 20 KU benzonase (Millipore) for 30 min on ice. 2× Laemmli sample buffer (Bio-Rad) was added to both pellet and supernatant fractions, boiled 5 min at 100°C and then loaded on an SDS gel.

### Competitive growth assay

Cells were counted and equal numbers of MRC5 CSB KO and MRC5^GFP-RPB1^ UVSSA KO cells or MRC5 UVSSA KO and MRC5^GFP-RPB1^ CSB KO cells were mixed together and their ratio was determined by flow cytometry. The cells were then co-cultured and either mock-treated or UV-treated (2 J/m^2^) every 24 h for 10 days. Cells were collected before UV-irradiation every second day and the changes in ratios between GFP-RPB1-expressing and not expressing cell populations were analyzed by flow cytometry. Mock-treated samples were used to correct for differences in growth rates at each time point. The fold change was calculated by comparing the percentage of all cells in the GFP-positive subpopulation to those in the GFP-negative subpopulation in mock-treated versus UV-treated samples.

### Transcription recovery assay

Cells were grown on 18 mm glass coverslips and mock-treated, irradiated with UV (8 J/m^2^) or treated with 40 ng/ml Illudin S for 3 h followed by a PBS wash, 18 h and 2 h (for UV) or 22 h and 2 h (for Illudin S) prior to addition of 200 μM 5′ethynyl uridine (EU, Jena Bioscience). Nascent RNA was labeled with EU for 1 h in Ham's F10 (Invitrogen) + 1% PS + 10% dialyzed FCS supplemented with 20 mM HEPES buffer (Lonza). Cells were fixed in 3.6% Formaldehyde for 15 min and permeabilized in 0.1% Triton X-100 in PBS for 10 min. Cells were blocked in 1.5% BSA in PBS for 10 min and EU was fluorescently labelled via click-it chemistry for 1 h by incubation with 50 mM Tris, 60 μM Atto-Azide-594 nm (Atto-tech), 4 mM CuSO_4_*5H_2_O (Sigma) and 10 mM ascorbic acid (Sigma). After labelling cells were washed 3× in 0.1% Triton X-100 for 5 min, washed 2× in PBS and then DNA was stained with 100 ng/ml DAPI (Sigma) in PBS for 20 min to visualize nuclei. Finally, cells were washed 2× in PBS and mounted on glass slides using Aqua-Poly/Mount (Polysciences). Fluorescent images were acquired on a Zeiss LSM700 microscope using a 1.3 NA 40× oil-immersion lens and ZEN software. Image analysis was performed in ImageJ and DAPI signal was used to determine the nuclei of cells and thereby the region to measure EU signal. The integrated density of each UV-treated cell was normalized to the average integrated density of mock-treated cells, which was set at 1.

### Fluorescence recovery after photobleaching (FRAP)

FRAP was performed on a Leica SP5 confocal microscope (for GFP-RPB1 expressing cells) or Leica SP8 (for CSB-mScarletI) using a HCX PL APO CS 63×, 1.40NA oil-immersion lens and LAS AF software. Fluorescence was detected using a 488 nm argon laser (GFP-RPB1) or 561 nm laser (CSB-mScarletI). A 512 × 32 pixel (GFP-RPB1) or 512 × 16 pixel-sized (CSB-mScarletI) strip was bleached across the nucleus of the cells using 100% laser power at 400 Hz for 1 (GFP-RPB1) or 2 frames (CSB-mScarletI). Fluorescence was measured at intervals of 0.4 s for 25 frames pre-bleach and 450 frames post-bleach (GFP-RPB1) or 5 frames pre-bleach and 40 frames post-bleach (CSB-mScarletI). The fluorescence intensity of the nucleus in the bleached strip was background corrected to the pre-bleach fluorescence intensity outside of the nucleus within the same strip. Relative Fluorescence Intensity (RFI) was calculated by normalizing fluorescence values post-bleach to average values pre-bleach, which was set at 100. Immobile fractions (*F*_imm_) were calculated by comparing the UV-irradiated conditions to the mock-treated condition for each cell line using the following formula:


\begin{equation*}{F}_{imm} = 1 - \frac{{\left( {{I}_{recovery,UV} - {I}_{bleach,UV}} \right)}}{{\left( {\left\langle {{I}_{recovery,untreated}} \right\rangle - {I}_{bleach,UV}} \right)}}\end{equation*}

where *I*_recovery,UV_ is the average RFI of frames 250–450 post-bleach (GFP-RPB1) or frames 24–33 post-bleach (CSB-mScarletI) of each UV-treated cell, *I*_bleach,UV_ is the RFI directly after bleaching and < *I*_recovery,untreated_> is the average RFI of frames 250–450 post-bleach (GFP-RPB1) or frames 24–33 post-bleach (CSB-mScarletI) of all cells in the untreated control cells.

### Flow cytometry

Cells were grown in 6-well plates and one 6-well was used per condition. When indicated, cycloheximide (100μM) was added to the cells 2 h prior to DNA damage induction (8 J/m^2^ UV-irradiation or 250 ng/ml Illudin S treatment for 24 h) and kept on the cells for the duration of the experiment to prevent new protein synthesis. A mock-treated sample (only cycloheximide) was included for each time/condition to correct for GFP-RPB1 decay over time. Cells were harvested by trypsinization, washed in PBS and fixed in 1% formaldehyde in PBS. Fluorescence levels were analyzed on an LSRFortessa Cell Analyzer (BD) equipped with FACSDiva Software (BD) and 10.000–20.000 cells were recorded per experiment. After exclusion of dead cells by gating for live cells based on granularity (SSC-A) and size (FSC-A) and doublet exclusion based on FSC-H versus FSC-W and SSC-H versus SSC-W gating, GFP levels were detected using a 488 nm laser and 530/30 filter. Flow cytometry data was analyzed using FlowJo software (v.10.8.1) from BD Biosciences and used to determine the mean fluorescence intensity of GFP-RPB1. Fluorescence intensity was background-corrected using non-fluorescent MRC5 WT cells and normalized to mock-treated fluorescence, which was set to 1. For analysis of Pol II levels in cells transiently overexpressing DDB2, DDB2-mCherry transfected cells were separated into DDB2 expressing/not expressing based on mCherry signal. mCherry levels were recorded using a 561 nm laser and 610/20 filter. GFP-RPB1 fluorescence intensity in UV-treated DDB2-expressing and non-expressing cells were normalized to mock-treated DDB2-expressing and non-expressing cells, respectively, which were set to 1.

### Immunofluorescence

Cells were grown on 18 mm glass coverslips. Cells were treated with 100 μM cycloheximide 2 h prior to mock- or UV-treatment (8 J/m^2^) and incubated for 6 h more with cycloheximide. Cells were fixed in 3.6% formaldehyde for 15 min and permeabilized in 0.1% Triton X-100 in PBS for 10 min. After washing in PBS, cells were blocked in 1% BSA in PBS for 25 min. The Pol II-Ser2 primary antibody was diluted in 1% BSA in PBS and added to the cells. After 1 h incubation cells were washed quickly 3x and extensively for 10 min in PBS. The secondary antibody and 100 ng/ml DAPI (Sigma) were also diluted in 1% BSA in PBS and incubated together on the cells. After 45 min incubation cells were washed quickly 3× and extensively for 10 min in PBS and then mounted on glass slides using Aqua-Poly/Mount (Polysciences). Fluorescent images were acquired on a Zeiss LSM700 microscope using a 1.3 NA 40× oil-immersion lens and ZEN software. Image analysis was performed in ImageJ and DAPI signal was used to determine the nuclei of cells and thereby the region to measure Pol II-Ser2 signal. The integrated density of each UV-treated cell was normalized to the average integrated density of mock-treated cells, which was set at 1. Information on used antibodies (e.g. clones, suppliers and dilutions) can be found in Supplementary Tables S4 and S5.

### Western blot

Proteins samples (total extracts or cell fractions) were separated on Mini-PROTEAN TGX 4–15% precast gels (BIORAD) in 1X running buffer (1.44% w/v glycine, 0.3% w/v TRIS, 0.1% w/v SDS). Proteins were transferred onto an ethanol-activated PVDF membrane (0.45 μm, Merck Millipore) in 1X transfer buffer (0.3% w/v Tris, 1.45% w/v glycine, 20% ethanol) at 30 V for 15 h at 4°C. Membranes were blocked in 5% skim-milk in PBS and stained with primary antibodies in PBS for 2 h at RT or 4°C overnight. Membranes were washed 3 × 10 min in 0.05% PBS-T after each antibody incubation. Secondary antibodies were conjugated to IR-Dyes (Sigma), incubated 1–2 h at RT and detected using Odyssey CL or CLx infrared scanners (LiCor). Antibodies are listed in Supplementary Tables S4 and S5.

## Results

### Differential Pol II chromatin-binding upon DNA damage in WT, CS and UV^S^S cells

**Figure 1.**
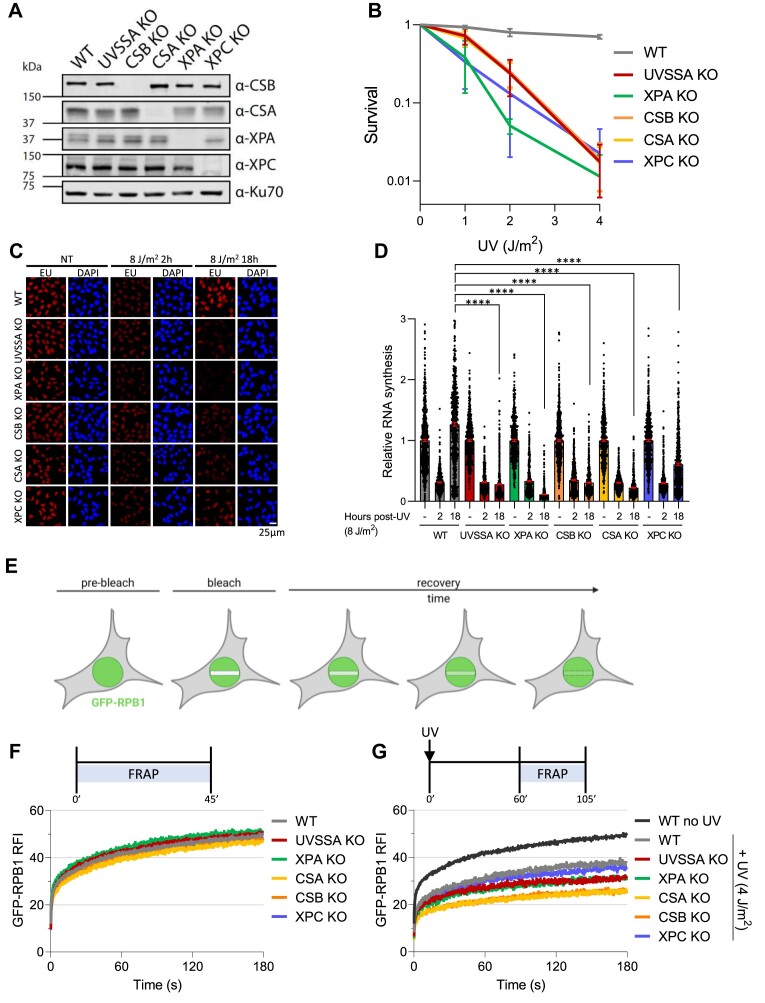
Different Pol II chromatin-binding dynamics after DNA damage observed in WT, CS and UV^S^S cells. (**A**) Western Blot analysis using the indicated antibodies of MRC5^GFP-RPB1^ WT cells and CRISPR/Cas9-mediated knock out (KO) cells of the indicated TC-NER proteins CSA and CSB, of the GG-NER protein XPC, and of the XPA protein involved in both sub-pathways. Ku70 was used as a loading control. Endogenously expressed UVSSA could not be detected and UVSSA KO was assessed by genotyping (Figure S1A). (**B**) Relative clonogenic survival of MRC5^GFP-RPB1^ WT or specified KO cells exposed to the indicated doses of UV (J/m^2^). ± SEM, *n* = 4. (**C**) Representative images of transcription levels as determined by relative EU incorporation in irradiated and non-irradiated cells 2 h and 18 h after UV exposure (8 J/m^2^). NT = non-treated. (**D**) Quantification of transcription restart after UV damage as determined by relative EU incorporation in MRC5^GFP-RPB1^ WT and indicated KO cells at the specified time points after UV exposure (8 J/m^2^) as shown in (C). The relative integrated fluorescence intensity was normalized to mock-treated levels and set at 1. Columns indicate average relative integrated fluorescence intensity and error bars ± SEM. *n* ≥ 504 cells per condition from at least four independent experiments. ****P< 0.0001. (**E**) Cartoon depicting Pol II Strip-FRAP procedure. GFP-RPB1 is bleached in a strip across the nucleus and recovery of fluorescence therein is monitored over time. (**F**) Fluorescence Recovery after photo-bleaching (FRAP) analysis of GFP-RPB1 under unperturbed conditions in WT or the indicated KO MRC5^GFP-RPB1^ cells. GFP-RPB1 was bleached and fluorescence intensity was measured every 0.4 sec for 3 min, background-corrected and normalized to pre-bleach fluorescence intensity, which was set to 100. Average Relative Fluorescence Intensity (RFI) of *n* ≥ 16 cells per condition from at least two independent experiments. (**G**) FRAP analysis similar as in (E), but 1–2 h after UV (4 J/m^2^) irradiation. GFP-RPB1 mobility of unperturbed WT cells is plotted in black for comparison. Average RFI of *n* ≥ 21 cells per condition from at least three independent experiments.

To test whether the phenotypic differences in CS and UV^S^S could be explained by differential chromatin-binding dynamics of Pol II upon TBL induction we employed Fluorescence Recovery after Photo-bleaching (FRAP) (52) of GFP-tagged Pol II, using knock-in (KI) cells expressing a GFP-tagged version of RPB1 at endogenous levels. FRAP of GFP-RPB1 KI cells is a sensitive tool to study the Pol II dynamics in living cells, at the different stages of the transcription cycle under unperturbed conditions (44) and upon DNA damage (21,45). We generated isogenic GFP-RPB1 KIs (MRC5^GFP-RPB1^) in WT and CSB, CSA, UVSSA, XPA and XPC knock-out (KO) cell lines (44–46). CSB and CSA KO cells represent CS cells, and UVSSA KO cells represent UV^S^S cells. XPC KO cells were included as a control for Global Genome NER (GG-NER) deficient cells, while XPA KO cells are deficient in both GG-NER and TC-NER. Correct KO of (TC-)NER genes was confirmed by genotyping using TIDE analysis (47) or by PCR (Supplementary Figure S1A–E), and absence of the (TC-)NER proteins was shown by western blot (Figure 1A). UVSSA KO was only confirmed by genotyping as endogenous UVSSA could not be detected by western blot. TC-NER deficiency of the KO cells was confirmed by clonogenic cell survival (Figure 1B and Supplementary Figure S1F) and by Recovery of RNA Synthesis after UV damage (Figure 1C, D). WT cells showed proficient colony formation and transcription restart. A similar UV-hypersensitivity and loss of transcription restart was observed in CSB, CSA and UVSSA KO cell lines. XPA KO cells showed an additional increase in UV-sensitivity as these cells are deficient in both GG-NER and TC-NER. Additionally, CSB, CSA, UVSSA and XPA KO cells were as expected hypersensitive to Illudin S, and showed no transcription restart upon exposure to this mushroom-derived toxin (Supplementary Figure S1G,H), which induces TBLs that are specifically recognized by TC-NER (53). XPC KO cells were as expected hypersensitive to UV due to their GG-NER deficiency, but showed transcription restart. Conversely, XPC KO cells were not Illudin S-sensitive, consistent with the requirement for TC-NER but not GG-NER in the repair of Illudin S-induced lesions. Collectively these data demonstrate a similar TC-NER deficiency in CSB, CSA and UVSSA KO cells.

Next, we used FRAP of GFP-RPB1 to compare Pol II chromatin binding kinetics under unperturbed conditions or after inducing TBLs by UV irradiation (Figure 1E). As promoter-bound Pol II complexes are chromatin-bound on average for less than a minute, they mostly contribute to the fast, initial recovery of fluorescence (<50 s) during GFP-RPB1 FRAP. In contrast, elongating Pol II complexes are chromatin-bound for >20 min on average, and are therefore mainly represented by the later part of the GFP-RPB1 FRAP curve (>100 s) (44). GFP-RPB1 FRAP analysis showed highly similar Pol II chromatin-binding kinetics in WT and the different KO cells without DNA damage induction (Figure 1F and Supplementary Figure S1I), indicating that transcription is not affected under unperturbed conditions in these KO cells. Irradiation with a relatively low dose of 4 J/m^2^ UV significantly reduced Pol II mobility in WT cells, evident from the reduced fluorescence recovery compared to unperturbed cells (Figure 1G and Supplementary Figure S1J). This immobilization affected both the initial part of the FRAP curve (<50 s) as well as the later part, evident from the reduced slope of the FRAP curve at time points >100 s. In line with previous findings (45), this can be explained by (i) degradation of promoter-bound Pol II which happens in a TC-NER independent manner and by (ii) stalling of elongating Pol II at TBLs. As expected, TC-NER proficient XPC KO cells, showed Pol II chromatin-binding dynamics comparable to WT cells. In TC-NER deficient CSB, CSA, UVSSA and XPA KO cells, however, Pol II showed a reduced mobility compared to WT and XPC KO cells. This was especially evident by the reduced slope of the FRAP curve at the later part of the curve, mainly representing elongating Pol II, suggesting that Pol II elongation rates were reduced, most likely caused by Pol II stalling at DNA damage. Interestingly, CSA and CSB KO cells showed a bigger immobile Pol II fraction upon UV damage compared to UVSSA and XPA KO cells (Figure 1G). Together these data suggest that either elongating Pol II is bound for a longer time, or more elongating Pol II is chromatin-bound, in the absence of CSB or CSA. This implies that CSA and CSB contribute to the release of lesion-stalled Pol II in the absence of TC-NER.

### Elongating Pol II is longer bound to TBLs in the absence of CSA and CSB

**Figure 2.**
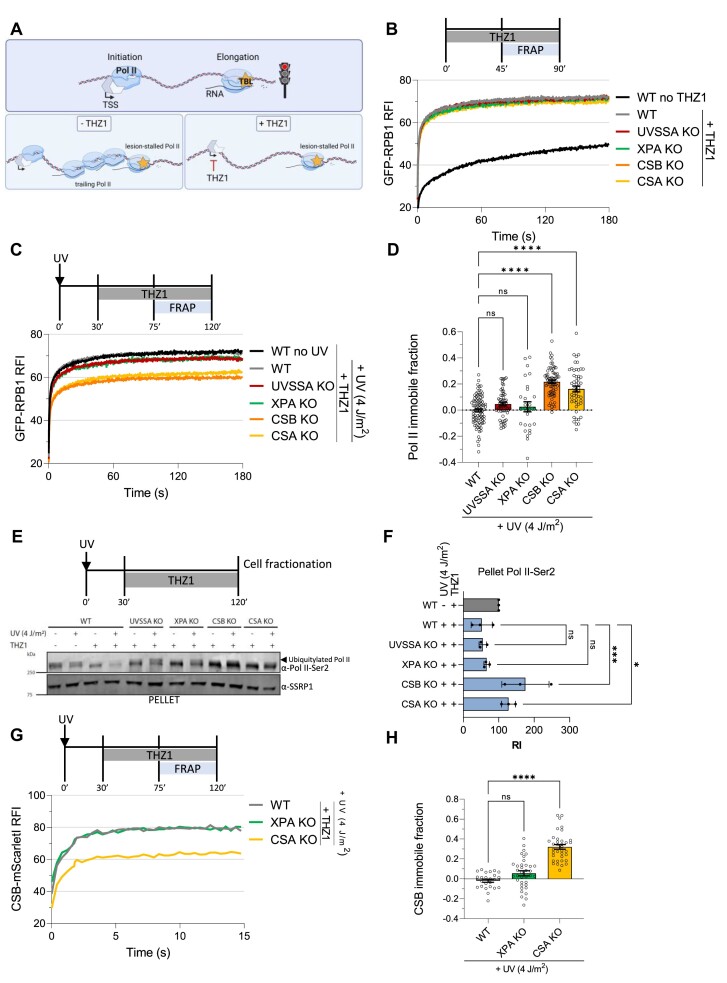
Prolonged chromatin-binding of elongating Pol II upon DNA damage in CSB and CSA KO cells. (**A**) Cartoon of experimental setup to study the residence time of lesion-stalled Pol II at TBLs. Elongating Pol II was allowed to run into UV-induced lesions for 30 min after which the *de novo* transcription initiation was blocked by the CDK7 inhibitor THZ1. This reduced the stalling of elongating Pol II molecules behind lesion-stalled Pol II in a ‘traffic jam’. This allowed us to mainly study lesion-stalled Pol II chromatin binding, as 45 min after CDK7 inhibition most elongating Pol II will be released from the chromatin due to transcription termination under unperturbed conditions. TSS = transcription start site. (**B**) Chromatin binding of Pol II as determined by FRAP of GFP-RPB1 in the indicated MRC5^GFP-RPB1^ WT and TC-NER-deficient cell lines under unperturbed conditions, and upon THZ1 treatment. Average Relative Fluorescence Intensity (RFI) as calculated by normalizing post-bleach values to pre-bleach values which were set at 100. RFI of *n* ≥ 29 cells per condition from at least 3 independent experiments. (**C**) FRAP analysis of GFP-RPB1 similar to (B), but after UV (4 J/m^2^) irradiation 30 min prior to THZ1 treatment. GFP-RPB1 mobility of unperturbed WT cells is plotted in black for comparison. Average RFI of *n* ≥ 27 cells per condition from at least three independent experiments. (**D**) Quantification of GFP-RPB1 immobile fraction after DNA damage obtained from (B, C). Immobile fraction in WT and TC-NER KO cells was calculated by comparing RFI after UV to mock-treated sample and normalizing to their own non-irradiated RFI values (See methods for more details). n ≥ 27 cells per condition from at least 3 independent experiments ± SEM. ns = not significant, ****P< 0.0001. (**E**) Representative western blot of pellet fraction after cell fractionation in MRC5^GFP-RPB1^ WT and indicated TC-NER KO cells. CTD-Ser2-phosphorylated RPB1 (Pol II-Ser2) staining in chromatin-fraction under unperturbed and UV-irradiated (4 J/m^2^) conditions upon THZ1 treatment to quantify chromatin-bound elongating Pol II. Higher-migrating Pol II-Ser2 band represents ubiquitylated elongating Pol II as indicated by arrow. SSRP1 is used as loading control. (**F**) Quantification of Pol II-Ser2 signal from three independent experiments as in (E). Pol II-Ser2 signal was normalized to SSRP1 (loading control) signal and to the mock-treated sample for each cell line and set at 100. RI = Relative intensity, ns = not significant, *P< 0.05, ***P< 0.001, ± SEM, *n* = 3. (**G**) FRAP of CSB-mScarletI in HCT116^CSB-mScarletI^ WT and TC-NER KO cells upon UV irradiation (4 J/m^2^) followed by THZ1 treatment. CSB-mScarletI was bleached in a strip across the nucleus and fluorescence intensity was measured every 0.4 s for 16 s 0–30 min after UV irradiation with the indicated UV doses. *n* ≥ 24 cells per condition from at least 2 independent experiments. (**H**) Quantification of CSB-mScarletI immobile fraction after DNA damage obtained from (G, S2E). Immobile fraction was calculated as in (D). *n* ≥ 24 cells per condition from at least 2 independent experiments ± SEM. ns = not significant, ****P< 0.0001.

Elongating Pol II molecules that are trailing, or halted, behind a lesion-stalled Pol II, may for a large part conceal the difference in residence times of lesion-stalled Pol II complexes in the different TC-NER KO cells. This is especially important since the number of lesion-stalled Pol II complexes will be rather small compared to the total number of elongating Pol II molecules at the relatively low, but physiologically relevant damage loads used in this study. To circumvent this, we developed an approach to more specifically study the residence time of lesion-stalled Pol II. To do so, after DNA damage infliction, we blocked *de novo* transcription initiation using the CDK7 inhibitor THZ1 (54) to reduce the number of elongating Pol II molecules trailing behind lesion-stalled Pol II (Figure 2A). We found that transcription inhibition for 45 min resulted in an almost complete mobilization of Pol II under undamaged conditions (Figure 2B and Supplementary Figure S2A). The increased Pol II mobilization can be explained by the accumulation of freely diffusing, i.e. highly mobile, Pol II, caused by a loss of immobile, chromatin-bound elongating Pol II. The immobile Pol II fraction is lost due to transcription termination at the end of genes in combination with a block of *de novo* transcription initiation (44). The Pol II mobilization upon THZ1 treatment under unperturbed conditions happened to similar extents in WT and TC-NER KO cells (Figure 2B). To study the residence time of lesion-stalled Pol II, we first UV irradiated the cells and allowed 30 min for elongating Pol II to encounter a lesion, thereafter *de novo* transcription initiation was inhibited by THZ1. THZ1 treatment in UV-irradiated WT cells resulted in a full mobilization of elongating Pol II similar to unperturbed conditions (Figure 2C and Supplementary Figure S2B). This indicates that under these conditions lesion-stalled Pol II is efficiently resolved, most likely due to TC-NER. In contrast, the Pol II immobile fraction was increased in UV-irradiated CSB and CSA KO cells compared to WT cells (Figure 2C, D and Supplementary Figure S2B), showing that 2 h after DNA damage induction ∼10–20% of all Pol II molecules in these cells remained stalled at a TBL. While a prolonged stalling of only 10–20% of all Pol II complexes may seem like a rather mild consequence, with ∼50.000 Pol II molecules present in the cell (44), these represent ∼5.000–10.000 large macromolecular chromatin-bound complexes that will interfere with most DNA transacting processes in the gene bodies. Interestingly, THZ1 treatment following UV-induced DNA damage in XPA and UVSSA KO cells led to only a very minimal immobilization, with Pol II TBL-binding kinetics mostly resembling those of WT cells (Figure 2C,D and Supplementary Figure S2B). This indicates that within this 2 h time-period most lesion-stalled Pol II is removed from the chromatin, even in the absence of TC-NER. Furthermore, this finding suggests that the intermediate Pol II immobilization observed without THZ1 upon UV-irradiation in XPA and UVSSA KO cells (Figure 1G) is mainly caused by elongating Pol II molecules that are trailing behind the initially stalled Pol II, which will subsequently stall on the same, unrepaired lesion due to the absence of TC-NER.

To confirm that elongating Pol II is chromatin-bound for a prolonged time upon DNA damage induction in CSA and CSB KO cells, we performed cell fractionation experiments followed by western blot analysis of RPB1. Chromatin-bound elongating Pol II was quantified by staining of serine 2-phosphorylated RPB1 (Pol II-Ser2) in the chromatin fraction (45). As expected, THZ1 treatment resulted in a reduction of elongating Pol II, in the absence of DNA damage. Levels of elongating Pol II were slightly further reduced upon TBL-induction (Figure 2E, F), which could be explained by Pol II release from the chromatin, as total levels of chromatin-bound Pol II were also reduced in this setting (Supplementary Figure S2C, D). In line with our FRAP results, a similar reduction in chromatin-bound Pol II-Ser2 comparable to WT was found in UVSSA and XPA KO cells upon DNA damage induction. In contrast, Pol II-Ser2 staining showed increased Pol II chromatin-binding in CSB and CSA KO cells upon TBL induction, confirming the prolonged stalling of Pol II in these cells. Interestingly, upon UV damage in UVSSA and XPA KO cells as well as in WT cells, slower migrating Pol II-Ser2 positive bands were detected, most likely representing ubiquitylated Pol II (34). These slower migrating bands were absent in CSA and CSB KO cells, explained by the absence of CRL4^CSA^ E3 ligase activity in these cells.

In cells, lesion-stalled Pol II is specifically recognized by CSB, resulting in a stable CSB-Pol II interaction (13–15). Therefore, CSB chromatin-binding, as determined by FRAP in CSB-mScarletI KI cells, can be used as a sensitive measure for lesion-stalled Pol II (21,48). To study CSB chromatin-binding dynamics in TC-NER deficient cells, we used isogenic KOs of CSA and XPA in HCT116^CSB-mScarletI^ cells (48). UVSSA KO cells were not used in this assay, as UVSSA is known to be crucial to stabilize CSB upon DNA damage by the recruitment of USP7 (24,25,48). No differences in CSB mobility upon THZ1 treatment in the absence of DNA damage were detected between WT and TC-NER KO cells (Supplementary Figure S2E). Next, we tested CSB chromatin-binding upon UV irradiation followed by THZ1 treatment and observed no immobilization in WT cells, which can be explained by TC-NER mediated TBL removal. Similar results were obtained in XPA KO cells, suggesting that, since CSB immobilization is a proxy for Pol II stalling at DNA damage, lesion-stalled Pol II is removed from the chromatin. In contrast, CSA KO cells exhibited significant CSB immobilization, indicative of prolonged stalling of Pol II on chromatin after DNA damage (Figure 2G,H). Taken together, these live cell imaging and cell fractionation assays show that Pol II is efficiently removed from TBLs in the absence of functional TC-NER in UVSSA and XPA KO cells. Conversely, in CSB and CSA KO cells lesion-stalled Pol II remains bound to chromatin for an extended period of time, underscoring the critical role of these proteins in the timely removal of lesion-stalled Pol II.

### Increased DNA damage-induced Pol II degradation in UVSSA-deficient cells

**Figure 3.**
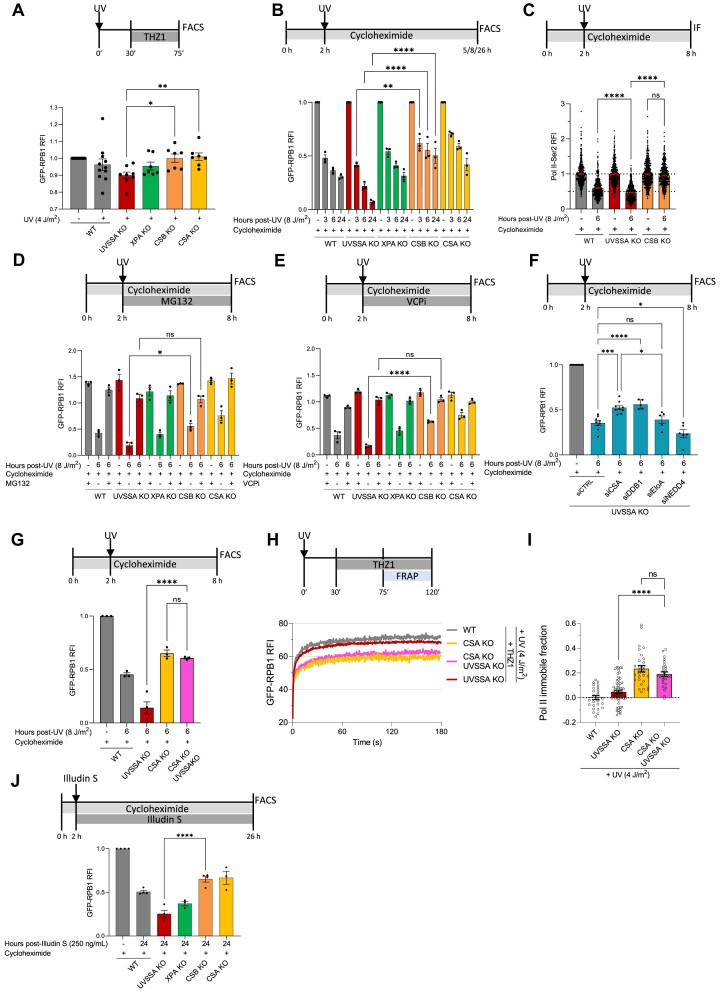
Absence of lesion-stalled Pol II degradation in CSA and CSB KO cells. (**A**) Pol II levels in MRC5^GFP-RPB1^ WT and indicated TC-NER cells were determined by measuring GFP-RPB1 intensity by flow cytometry (>10.000 cells). Cells were irradiated with UV (4 J/m^2^) 30 min prior to THZ1 treatment. GFP-RPB1 levels in UV-irradiated cells were normalized to non-damaged samples for each cell line that were set at 1. Columns represent average RFI of n ≥ 7 independent experiments ± SEM. *P< 0.05,**P< 0.01. (**B**) Pol II levels in the indicated MRC5^GFP-RPB1^ cells as in (A) at the indicated times after UV-irradiation (8 J/m^2^) in the presence of cycloheximide (100 μM). Cycloheximide was added 2 h prior to UV-irradiation and maintained for the duration of the experiment. UV-treated samples were corrected to non-damaged GFP-RPB1 levels of each cell line at each time point and normalized to sample treated for 5 h with cycloheximide which was set at 1. Columns represent average RFI of *n* = 3 independent experiments ± SEM. **P< 0.01, ****P< 0.0001. (**C**) Quantification of elongating Pol II levels by immunostaining using a Pol II-Ser2 antibody in cycloheximide-treated (as in B) MRC5^GFP-RPB1^ WT and indicated KO cells 6 h after UV exposure (8 J/m^2^). The relative integrated fluorescence intensity was normalized to mock-treated levels and set at 1. Columns indicate average relative integrated fluorescence intensity and error bars ± SEM. *n* ≥ 404 cells per condition from three independent experiments. ns = not significant, ****P< 0.0001. (**D**) Pol II levels as in (B) with the addition of proteasome inhibition with MG132 (50 μM) which was added at the time of UV-irradiation (8 J/m^2^). GFP-RPB1 levels in all samples were corrected and normalized to non-damaged samples treated 8 h with cycloheximide without proteasome inhibitor which were set at 1. Columns represent average RFI of n = 3 independent experiments ± SEM. *P< 0.05, ns = not significant. (**E**) Pol II levels as in (D) but now in the presence of VCP inhibition using NMS-873 (5 μM). *n* = 3 independent experiments ± SEM. ****P< 0.0001, ns = not significant. (**F**) Pol II levels in UVSSA KO cells as in (B) upon transfection of the indicated siRNAs and harvested 6 h post-irradiation (8 J/m^2^). siCTRL is a non-targeting siRNA used as control. UV-treated samples were normalized to non-damaged samples treated 8 h with cycloheximide which were set at 1. Columns represent average RFI of n ≥ 5 independent experiments ± SEM. *P< 0.05, ***P< 0.001, ****P< 0.0001, ns = not significant. (**G**) Pol II levels as in (F) in the indicated MRC5^GFP-RPB1^ cells. Columns represent average RFI of n ≥ 3 independent experiments ± SEM. ****P< 0.0001, ns = not significant. (**H**) FRAP of GFP-RPB1 in CSA KO and UVSSA/CSA double KO cells as described in (2C). GFP-RPB1 mobility of UV-irradiated UVSSA KO cells from (2C) is plotted in red for comparison. Average RFI of n ≥ 27 cells per condition from 3 independent experiments. (**I**) Quantification of GFP-RPB1 immobile fraction after DNA damage obtained from (H, S4F). Immobile fraction was calculated as in (2D). n ≥ 27 cells per condition from three independent experiments ± SEM. ns = not significant, ****P< 0.0001. (**J**) Pol II levels upon treatment with Illudin S (250 ng/ml) for 24 h and cycloheximide as in (B). Illudin S treated samples were normalized to non-damaged samples treated 26 h with cycloheximide which were set at 1. Columns represent average RFI of *n* ≥ 3 independent experiments ± SEM. ****P< 0.0001.

Next, we set out to study the underlying mechanism explaining how lesion-stalled Pol II is displaced from the chromatin in the absence of TC-NER in UVSSA and XPA KO cells. In UVSSA and XPA KO cells the CRL4^CSA^ E3 ligase is still recruited to lesion-stalled Pol II (16). As CRL4^CSA^ has been shown to stimulate the ubiquitylation of elongating Pol II upon DNA damage (18,34), Pol II may be degraded in UVSSA and XPA KO cells while in CSA and CSB KO cells this would not happen due to the loss of CRL4^CSA^ activity. To test this hypothesis, we assessed Pol II degradation by quantifying endogenously expressed GFP-RPB1 protein levels by flow cytometry (Supplementary Figure S3A, B), which is a sensitive approach to quantify the total cellular Pol II levels without any antibody or extraction bias (44). Under conditions in which new transcription initiation events are inhibited by THZ1, exactly as used in the FRAP experiments (Figure 2), UV-irradiation induced a minor Pol II degradation in WT cells, which was exacerbated in UVSSA KO, but completely absent in CSB and CSA KO cells (Figure 3A). Importantly, although the difference in Pol II levels in UVSSA KO compared to CSA and CSB KO cells seems rather small (∼10%), this is in line with the ∼10–20% immobilized Pol II observed in CSA and CSB KO cells (Figure 2D), suggesting that specifically the TBL-stalled Pol II fraction is removed from chromatin by degradation in UVSSA KO cells. Interestingly, while in XPA KO cells Pol II is also released from the chromatin similarly as in UVSSA KO cells, no increased Pol II degradation could be observed compared to WT cells. This indicates that in XPA KO cells Pol II is not degraded but is likely released in a reaction step downstream of UVSSA. XPA is recruited to the TBL after TFIIH binds to the damaged DNA, and Pol II is therefore most likely already removed from the TBL preceding this reaction step, either by backtracking or chromatin release (2). The difference in Pol II degradation in UVSSA and XPA KO cells, suggests that in the absence of TC-NER, Pol II can be released from damaged chromatin by two different mechanisms.

When studying Pol II degradation in the presence of THZ1, we mostly assessed the stability of the first elongating Pol II that would stall on a TBL, as consecutive rounds of Pol II stalling at unrepaired TBLs were prevented due to inhibition of transcription initiation. However, under conditions of normal transcription initiation it is expected that consecutive rounds of Pol II will stall at unrepaired TBLs, which might result in more Pol II degradation. Therefore, we quantified Pol II degradation over a period of 24 h after UV in the absence of THZ1. Under these conditions a ∼50% loss of Pol II was observed in WT cells (Supplementary Figure S3C). However, these measurements were obscured by increased Pol II levels 24 h after UV-irradiation, most likely due to *de novo* Pol II synthesis upon transcription restart after TBLs are repaired. To study specifically Pol II degradation without considering effects of *de novo* translation, protein synthesis was inhibited with the ribosome inhibitor cycloheximide, which under the used conditions did not to interfere with TC-NER (Supplementary Figure S3D, E). Under these conditions a gradual drop in Pol II levels upon UV damage was observed in time in WT cells (Figure 3B). A similar gradual Pol II degradation could be observed in XPA KO cells, confirming that in XPA KO cells Pol II is degraded to a comparable level. In CSA and CSB KO cells, Pol II degradation was much less pronounced (Figure 3B and Supplementary Figure S3F), which is consistent with the impeded degradation of lesion-stalled Pol II and in line with existing literature (18,34). In sharp contrast, we found a strongly increased Pol II degradation over time in UVSSA KO cells (Figure 3B and Supplementary Figure S3F), with almost no Pol II left after 24 h, which is likely caused by consecutive rounds of Pol II encountering unrepaired TBLs followed by its degradation.

This CSA and CSB-dependent loss of Pol II upon UV damage mostly represents elongating Pol II as shown by immunofluorescence staining of Pol II-Ser2, which showed a complete absence of degradation of elongating Pol II in CSB deficient cells 6 h after UV-induced damage (Figure 3C and Supplementary Figure S3G). Of note, under these conditions where *de novo* translation is absent, Pol II degradation is observed in all cell lines including the CSA and CSB KO cells, especially at the earliest measured time-point (3 h post-damage). However, this degradation does not represent elongating Pol II-Ser2 (Figure 3C and Supplementary Figure S3G). This indicates that in addition to the CSA and CSB dependent degradation of lesion-stalled Pol II (Figure 3A, C), also a TC-NER independent degradation of Pol II occurs. This could for example be attributed to the recently described degradation of promoter-bound Pol II, which happens shortly after UV-induced DNA damage independently of TC-NER (45,55)

As degradation of elongating Pol II correlates with the presence of the CRL4^CSA^ E3 ligase, this suggests that Pol II is being degraded in a ubiquitin and 26S proteasome dependent manner. To test this, we assessed Pol II levels upon proteasome inhibition using MG132 or Bortezomib. Proteasome inhibition rescued DNA damage-induced Pol II loss in the different TC-NER KO cells to a similar extent (Figure 3D and Supplementary Figure S3H), indicating that Pol II is being degraded by the 26S proteasome. Importantly, this also indicates that the additional loss of Pol II in UVSSA KO cells compared to CSA and CSB KO cells is caused by proteasomal degradation of lesion-stalled Pol II. As the extraction of chromatin-bound Pol II upon DNA damage has been described to depend on the ubiquitin selective segregase p97/VCP (45,56), we tested its involvement in this process. Similarly as upon proteasome inhibition, VCP inhibition by NMS-873 led to a comparable rescue of Pol II levels in all cell lines (Figure 3E), implying that in UVSSA KO cells lesion-stalled Pol II is ubiquitylated to be subsequently extracted from the chromatin by VCP and degraded. Therefore, we explored the contribution of the CRL4^CSA^ E3 ligase to the Pol II degradation observed in UVSSA KO cells, and compared this to the contribution of the Elongin A (EloA) and NEDD4 E3 ligase complexes, that have been described to ubiquitylate elongating Pol II as part of the last-resort Pol II degradation pathway (57). While depletion of NEDD4 or EloA did not significantly reduce Pol II degradation in UVSSA KO cells, depletion of CSA clearly reduced Pol II degradation compared to control conditions (Figure 3F and Supplementary Figure S3I). Nonetheless, the rescue of Pol II degradation achieved by siRNA-mediated CSA depletion in UVSSA KO cells was not to the same level as observed in CSA KO cells (Figure 3B), most likely due to an incomplete CSA knockdown. To test this we generated CSA/UVSSA double KO cells (Supplementary Figure S4A-E) and studied the Pol II degradation in in these cells. In CSA/UVSSA double KO cells Pol II degradation was fully rescued and indistinguishable from CSA KO cells (Figure 3G), indicating removal of lesion-stalled Pol II by CRL4^CSA^-mediated proteasomal degradation in UVSSA KO cells. In line with this notion, after UV-irradiation and THZ1-treatment Pol II remained bound to chromatin in CSA/UVSSA double KO cells at comparable levels to CSA KO cells (Figure 3H, I and Supplementary Figure S4F–H). To further pinpoint the need for the E3 ligase activity of the CRL4^CSA^ complex in Pol II degradation in UVSSA KO cells, we depleted the crucial DDB1 component of the CRL4^CSA^ complex (Supplementary Figure S3J). DDB1 depletion rescued Pol II degradation to a similar degree as CSA depletion (Figure 3F). Together, these findings indicate that the CRL4^CSA^ ubiquitin ligase activity is crucial for the removal of Pol II at TBLs in UV^S^S cells and that the sustained Pol II stalling associated with CS is the dominant phenotype.

In order to determine whether the differential Pol II degradation across TC-NER deficient cells is specific for UV-induced damage, or is also observed upon induction of other structurally different types of TBLs, we exposed cells to Illudin S. Treatment with Illudin S increased Pol II degradation in UVSSA KO cells compared to CSA and CSB KO cells, similarly as was observed after UV-induced damage (Figure 3J). Together this shows that Pol II stalled at different types of TBLs is not degraded in CSA and CSB KO cells, resulting in prolonged stalling of elongating Pol II on TBLs, while lesion-stalled Pol II is efficiently extracted from the chromatin by VCP resulting in its degradation in UVSSA KO cells.

### Residence time of lesion-stalled Pol II correlates with patient-specific mutations

**Figure 4.**
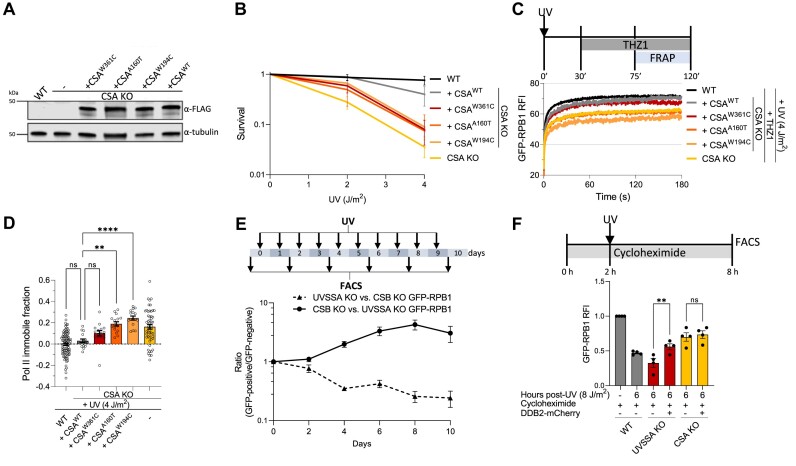
Pol II mobility correlates with patient-specific UV^S^S and CS mutations. (**A**) Western blot of MRC5^GFP-RPB1^ WT and CSA KO cells complemented with FLAG-tagged WT CSA or CSA harboring point mutations W361C, A160T or W194C. Exogenous expressed CSA is stained with FLAG antibody and tubulin is used as loading control. (**B**) Relative clonogenic survival of the indicated cell lines in response to the specified UV doses. ± SEM, *n* ≥ 3. (**C**) FRAP of GFP-RPB1 upon UV irradiation (4 J/m^2^) followed by THZ1 treatment in CSA KO cells reconstituted by expression of either the WT or the indicated CSA mutants. GFP-RPB1 mobility of UV-irradiated and THZ1-treated WT and CSA KO cells is plotted in black and yellow, respectively, for comparison. Average RFI of *n* ≥ 16 cells per condition from at least 2 independent experiments. (**D**) Quantification of GFP-RPB1 immobile fraction from (C, S4C). *n* ≥ 16 cells per condition from at least two independent experiments ± SEM. **P< 0.01, ****P< 0.0001, ns = not significant. (**E**) MRC5 CSB KO and MCR5^GFP-RPB1^ UVSSA KO cells or MRC5 UVSSA KO and MRC5^GFP-RPB1^ CSB KO cells were co-cultured and either mock- or UV-treated (2 J/m^2^) daily for 10 days. Changes in ratios between GFP-RPB1-expressing and not expressing cell populations were determined by flow cytometry every second day. Mock-treated samples were used to correct for differences in growth rates. Data points represent the average of three independent experiments and error bars ± SEM. (**F**) Pol II levels in the indicated MRC5^GFP-RPB1^ cells 6 h after UV-irradiation (8 J/m^2^) in the presence of cycloheximide (100 μM), added 2 h prior to UV-irradiation and maintained for the duration of the experiment. DDB2 was transiently overexpressed in UVSSA KO and CSA KO cells, and only DDB2 overexpressing cells were analyzed when indicated. UV-treated samples were normalized to mock-treated samples for each condition which was set at 1. Columns represent average RFI of *n* = 4 independent experiments ± SEM. **P< 0.01, ns = not significant.

To directly link the observed differences in UVSSA KO cells versus the CSA and CSB KO cells to the UV^S^S and CS disorders, we studied Pol II chromatin binding in cells expressing well-defined patient-specific CSA mutations that either give rise to CS or UV^S^S. We selected the W361C mutation causing UV^S^S (35) and two CS-causing CSA point mutations. W194C giving rise to CS-I, the classical severe form of CS, and A160T results in CS-III, a late onset form of CS with milder clinical features (29,41). Based on the CSA-DDB1 structure, the CS-causing W194C and A160T mutations are predicted to interfere with the CSA fold, which is more severely disrupted by the W194C mutation. Both mutations interfere with the binding of CSA to DDB1 resulting in the inactivation of the CRL4^CSA^ ligase activity (42). In line, A160T prevents the incorporation of CSA into the CRL4^CSA^ complex (49). In contrast, the UV^S^S-causing W361C mutation severely impairs UVSSA recruitment, while leaving the CRL4^CSA^ activity intact (35). FLAG-tagged WT CSA, or CSA harboring the patient-specific point mutations was expressed in MRC5^GFP-RPB1^ CSA KO cells (Figure 4A and Supplementary Figure S5A, B). The TC-NER status of the cell lines was confirmed by testing UV sensitivity by colony formation. Expression of WT CSA rescued the UV-sensitivity in CSA KO cells. However, expression of CSA mutants did not rescue the UV sensitivity observed in CSA KO cells, consistent with a TC-NER deficiency prevalent in CS and UV^S^S patients (Figure 4B).

Next, we performed Pol II FRAP to assess Pol II chromatin binding in the cells expressing these patient-specific CSA mutants. Under undamaged conditions no differences in Pol II chromatin binding were observed in the different CSA mutant expressing cell lines (Supplementary Figure S5C, D). Upon DNA damage induction followed by THZ1 treatment, Pol II chromatin binding in CSA^WT^ expressing cells was similar to WT cells, indicative of a full rescue of the prolonged Pol II stalling in CSA KO cells. In line with our hypothesis, CSA^W361C^ expressing cells showed a very similar Pol II mobility as CSA^WT^ expressing cells, while Pol II was markedly immobilized in CSA^A160T^ and CSA^W194C^ expressing cells, to nearly the same level as in CSA KO cells (Figure 4C, D and Supplementary Figure S5E). By using mutations in the same protein either causing CS or UV^S^S we thus confirm that in UV^S^S cells, lesion-stalled Pol II is resolved, while the prolonged stalling of Pol II at TBLs is a specific feature of CS cells, and therefore contributes to the severe CS phenotypes.

**Figure 5.**
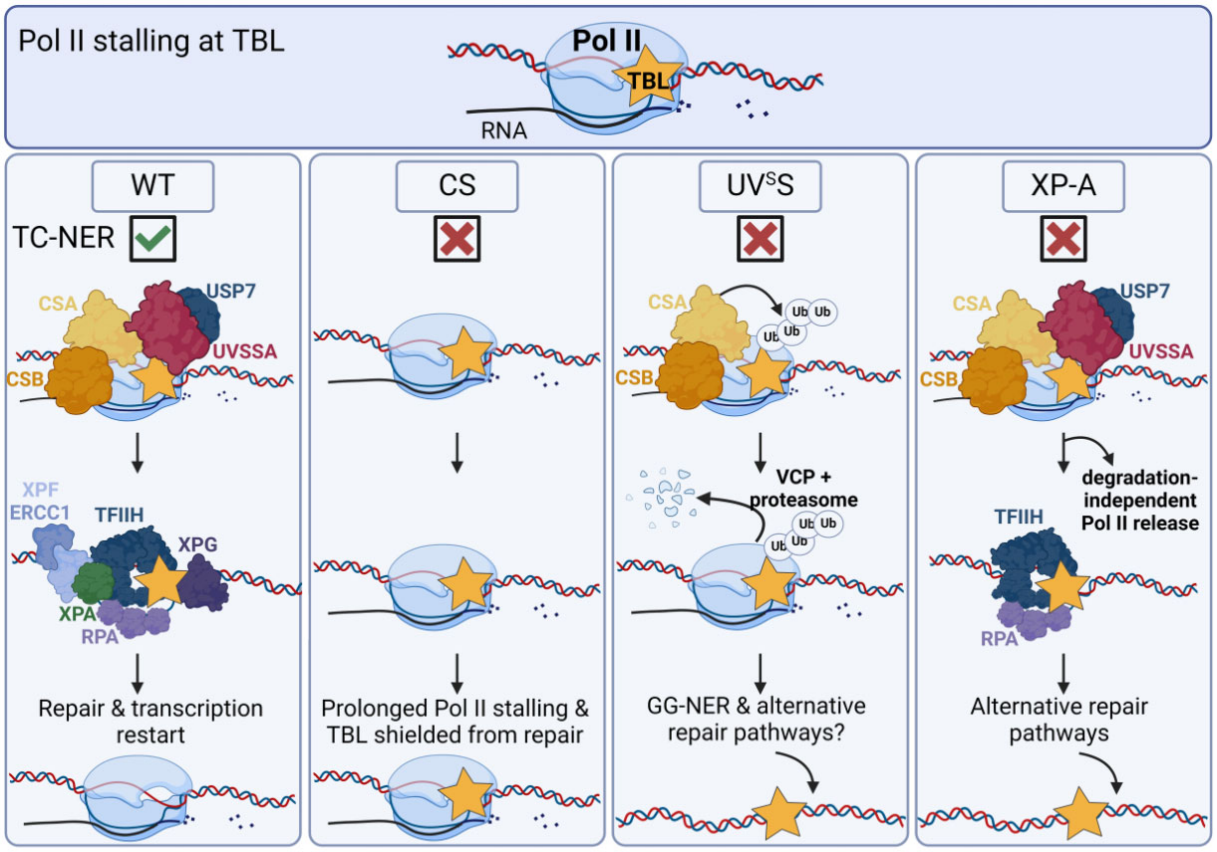
Model showing the fate of Pol II in WT, CS, UV^S^S, and XP-A cells. When elongating Pol II encounters a TBL it can have different outcomes. In WT and XP-A cells Pol II will be removed from the TBL during the repair reaction to allow repair of the lesion via TC-NER without being extensively degraded. However, in XP-A cells TC-NER cannot be completed, but the lesion will be accessible for alternative repair pathways. In UV^S^S cells Pol II will be ubiquitylated in a CRL4^CSA^-dependent manner, removed from the lesion by the ubiquitin-specific protease VCP and subsequently degraded by the 26S proteasome, thereby exposing the lesion to other repair pathways that can eventually repair the damage. In CS cells Pol II will remain bound to the TBL creating a highly toxic persistent transcription block.

Our model suggests that the milder clinical phenotype of UV^S^S most likely can be explained by the degradation of Pol II (Figure 5), which might allow access of alternative repair mechanisms to the TBL (2). A likely backup repair pathway of UV-induced TBLs is GG-NER. While, GG-NER efficiently repairs 6–4PPs, UV-induced CPD lesions are repaired relatively slowly by GG-NER due to low expression levels of DDB2 (58,59). This most likely explains why no differences are observed between CS and UV^S^S cells in clonogenic survival assays (Figure 1B). However, such differences in DNA damage tolerance between CS and UV^S^S cells might become apparent when exposed to low levels of UV damage over an extended period of time in competitive growth assays, which are sensitive to detect small differences in sensitivity to DNA damage. Therefore, we co-cultured UVSSA and CSB KO cells, in which either the UVSSA or the CSB KO cells were GFP-positive, and cells were irradiated with a low UV dose of 2 J/m^2^ over a period of 10 consecutive days. Changes in the ratios between these two TC-NER deficient cell populations were assessed by flow cytometry. While in undamaged conditions no distinct differences in growth could be observed (Supplementary Figure S6A), the UVSSA KO cells outcompeted CSB KO cells upon UV-damage induction (Figure 4E and Supplementary Figure S6A).

Next, we addressed the contribution of GG-NER to mitigate DNA damage-induced transcription stress in UV^S^S cells. By overexpressing DDB2 in UVSSA KO cells CPD repair by GG-NER was enhanced (59). TBL repair by GG-NER in UVSSA KO cells would prevent consecutive rounds of Pol II stalling and subsequent degradation on the same UV-lesion, thereby reducing the extensive Pol II degradation observed in UVSSA KO cells. Indeed, overexpression of DDB2 in UVSSA KO cells significantly reduced Pol II degradation compared to UVSSA KO cells that did not overexpress DDB2 (Figure 4F and Supplementary Figure S6B). In line with such a DDB2-mediated repair of CPDs in UVSSA KO cells, the rescue of Pol II levels fully depended on GG-NER, as DDB2 overexpression had no effect on Pol II degradation in UVSSA/XPC double KO cells (Supplementary Figure S6C-G). DDB2 overexpression did not affect Pol II degradation in WT (Supplementary Figure S6H) or CSA KO cells (Figure 4F), suggesting that increased GG-NER activity only has an effect on Pol II levels when the unrepaired lesion is made accessible by degradation of lesion-stalled Pol II. Collectively, these experiments suggest an important role for GG-NER as a backup repair pathway in UVSSA KO cells, when the lesion is made accessible by CRL4^CSA^-mediated Pol II degradation.

## Discussion

This study shows that Pol II is differentially extracted from chromatin upon DNA damage in CS and UV^S^S cells and that the impaired extraction of Pol II from TBLs is associated with increased severity of TC-NER related syndromes. We show that live cell imaging mobility studies of Pol II, following inhibition of transcription initiation, is a powerful setup to study the residence time of lesion-stalled Pol II. Using this approach, we observed a prolonged binding of Pol II to UV-damaged chromatin in CS cells, while Pol II was released from the chromatin in UVSSA and XPA KO cells to a similar degree as in TC-NER proficient cells. At the relatively low UV dose used, a fraction of 10–20% of all Pol II molecules is chromatin-bound at DNA damage in CS cells, representing 5.000–10.000 Pol II complexes that will form long-lasting roadblocks for transcription, thereby inactivating many genes. Additionally, these roadblocks will most likely impede other DNA transacting processes, or will result in transcription-replication conflicts, an important source of genome-instability (60).

Thus far the degradation of elongating Pol II upon DNA damage was under debate. Some studies showed that UV-induced Pol II degradation was absent in CS patient cells (19,34), while other studies observed Pol II ubiquitylation and degradation in CSB and CRL4^CSA^ deficient cells (20,61). Here we show that in isogenic TC-NER deficient cells the degradation of lesion-stalled Pol II is almost completely absent in CS cells, in line with the lost CRL4^CSA^ activity in these CS cells. In contrast, in UVSSA KO cells Pol II is extensively degraded, suggesting that almost all lesion-stalled Pol II is released from chromatin by degradation in these cells. Importantly, we show that the Pol II degradation in UVSSA KO cells is mainly caused by CRL4^CSA^ activity, while only a negligible contribution to Pol II degradation was observed for the E3 ligases NEDD4 and EloA, which have been suggested to coordinate Pol II degradation as a last resort mechanism (57,61,62). Also BRCA1-BARD1 (63) and VHL (56,64,65) were linked to Pol II ubiquitylation upon DNA damage. The existence of these different E3 ubiquitin ligases underlines the importance of Pol II degradation to warrant transcription integrity. Future research should uncover the precise mechanism and under which exact conditions, e.g. limiting TC-NER conditions, specific types of TBLs or specific tissues or cell types these E3 ligase are involved.

Here we experimentally confirm and extend earlier proposed models (2,34) thereby explaining phenotypical differences between CS and UV^S^S (Figure 5), in which Pol II stalling at TBLs happens in a similar manner irrespective of the TC-NER status, but in which the subsequent Pol II processing happens differently. In WT cells the majority Pol II is released from the TBL through repair by TC-NER, without significant Pol II degradation. Whether Pol II is released from the chromatin during the repair reaction, or is back-tracked and subsequently released upon transcription termination at the end of the gene upon repair, remains an important open question in the TC-NER field. In the absence of UVSSA, Pol II TBL-binding is not sustained despite defective TC-NER as Pol II still can be ubiquitylated by the CRL4^CSA^ E3 ligase. Ubiquitylated Pol II will be recognized by VCP, extracted from chromatin and subsequently degraded by the 26S proteasome. As a consequence, the lesion is made accessible to alternative repair factors. We showed that GG-NER is an important alternative repair pathway in UVSSA KO cells, able to reduce excessive Pol II degradation in these cells, which is a hallmark of DNA damage-induced transcription stress (66). GG-NER efficiently recognizes and removes UV-induced 6–4-PPs. However, GG-NER is inefficient at recognizing CPDs (67), possibly explaining the cutaneous photosensitivity of UV^S^S patients. When CSB or CSA are defective, the CRL4^CSA^ E3 ligase complex fails to ubiquitylate lesion-stalled Pol II, leading to a prolonged association of Pol II to the lesion and a complete shielding of UV-induced TBLs from other repair processes like GG-NER (3). In time, due to accumulating DNA damage, this will result in a gradual increase of lesion-stalled Pol II complexes. In XPA-deficient cells a similar Pol II degradation to that of TC-NER proficient cells was observed, consistent with the idea that Pol II has already been backtracked or released from the chromatin at the time of XPA recruitment, which happens during the TFIIH-mediated proofreading and excision step of TC-NER. However, while the lesion is therefore most likely accessible due to Pol II release, both TC-NER and GG-NER are impaired, thus eliminating the main pathways for removal of bulky DNA lesions. Only lesions that are not substrates for NER can still be repaired by other pathways, such as Base Excision Repair (BER) that repairs oxidative damage. This might explain the phenotype of the XPA-associated Xeroderma pigmentosum (XP) patients with mostly milder neurological and clinical phenotypes (68).

Our model also explains the striking differences in disease severity observed for different mutations in a single gene, as is the case for CSA. Mutations such as CSA^A160T^ and CSA^W194C^, affecting the E3 ligase function, prevent Pol II release from chromatin and therefore cause a more severe phenotype. On the other hand, the CSA^W361C^ mutation, interfering with the recruitment of UVSSA, but leaving the CRL4^CSA^ activity intact, will allow ubiquitylation and extraction of lesion-stalled Pol II and thus result in a milder disease phenotype. This model furthermore predicts that mutations in other TC-NER factors that impede Pol II ubiquitylation will result in CS-like phenotypes. In line with this, a mouse model in which the K1268 ubiquitylation site of Pol II is mutated features a CS-like phenotype, including growth retardation, progressive neurodegeneration and early death (18). Collectively these insights show that the ability to remove Pol II from the lesion is an important determinant for disease outcome, and indicate that these unresolved lesion-stalled Pol II complexes are more detrimental for the cell than the residual TBL itself. Interestingly, such a link between the accumulation of persistent, damage-bound repair intermediates and neurodegeneration was also shown for TFIIH, which in the absence of the endonucleases ERCC1-XPF or XPG is bound for an extended period, resulting in functional impairment of differentiated neurons *in vivo* (69).

While transcription is an essential process in every cell, it is interesting to note that CS patients are especially characterized by extreme neurological dystrophy. Due to the post-mitotic nature of neurons, other mechanisms that could result in clearing of lesion-stalled Pol II, such as DNA replication-mediated Pol II clearance, will not be active. Alternatively, neurons might be particularly sensitive to DNA damage-induced transcription stress because they depend on expression of relatively long neuronal genes (67). Such long neuronal genes have a high likelihood of accumulating DNA damage that will block transcription. Obviously, neurodegeneration associated with CS and XPA-deficiency can hardly be explained by the prevalence of UV-induced CPDs and 6-4-PPs. The type of damage that causes neurodegeneration in NER disease is currently not exactly known, but aldehydes, alkylating agents or reactive oxygen species (ROS) (70,5) are likely candidates to contribute to the accumulation of DNA damage in neurons. Based on our model, most likely only TBLs that are inaccessible for alternative repair pathways, due to the size and placement of Poll II, will lead to Pol II accumulation on transcribed genes over time and will require CRL4^CSA^-dependent Pol II degradation. Other obstructions such as macromolecular complexes crosslinked to the chromatin, including large protein-DNA crosslinks (DPCs), will presumably not be completely shielded by the elongating Pol II and may therefore be removed without the need to release Pol II by degradation.

Interestingly, recent studies have uncovered that these large DPCs are repaired by a dedicated transcription-coupled repair pathway (TC-DPC repair). In TC-DPC repair transcription-blocking DPCs are repaired by a non-canonical TC-NER mechanism, in which the DPC is degraded by CRL4^CSA^ activity, in a mechanism independent of downstream TC-NER factors like UVSSA and XPA (46,71,72). These findings therefore suggest an additional explanation for the observed phenotypic differences between CS and UV^S^S (46). While at first glance the mechanisms of TC-DPC repair and Pol II degradation seem very different, they are actually very much alike, while in TC-DPC repair the DPC is ubiquitylated by CRL4^CSA^ to be subsequently degraded, in UV^S^S cells the sustained chromatin-binding of Pol II at DNA damage resembles a DPC and is also ubiquitylated by the same E3 ubiquitin ligase complex. These data show the importance of future studies to better understand the types(s) of endogenous DNA damage involved in TC-NER syndromes and the implicated repair mechanisms.

In conclusion, we find that specifically CS cells are defective in Pol II clearance, resulting in the accumulation of TC-NER intermediates that prevent alternative pathways from accessing the DNA damage. Our findings demonstrate that it is not the TC-NER defect itself but rather the accumulation of Pol II intermediates that contributes to the neurodegenerative phenotype of CS.

## Supplementary Material

gkae618_Supplemental_File

## Data Availability

The data underlying this article will be shared on reasonable request to the corresponding author.
