# Supplementary material for: Differential processing of RNA polymerase II at DNA damage correlates with transcription-coupled repair syndrome severity
Source: Nucleic Acids Res. 2024 Jul 18;52(16):9596–612. doi: 10.1093/nar/gkae618 (PMC11381366; doi:10.1093/nar/gkae618)
Supplement: gkae618_Supplemental_File [file gkae618_supplemental_file.pdf]

Supplementary Figure S1

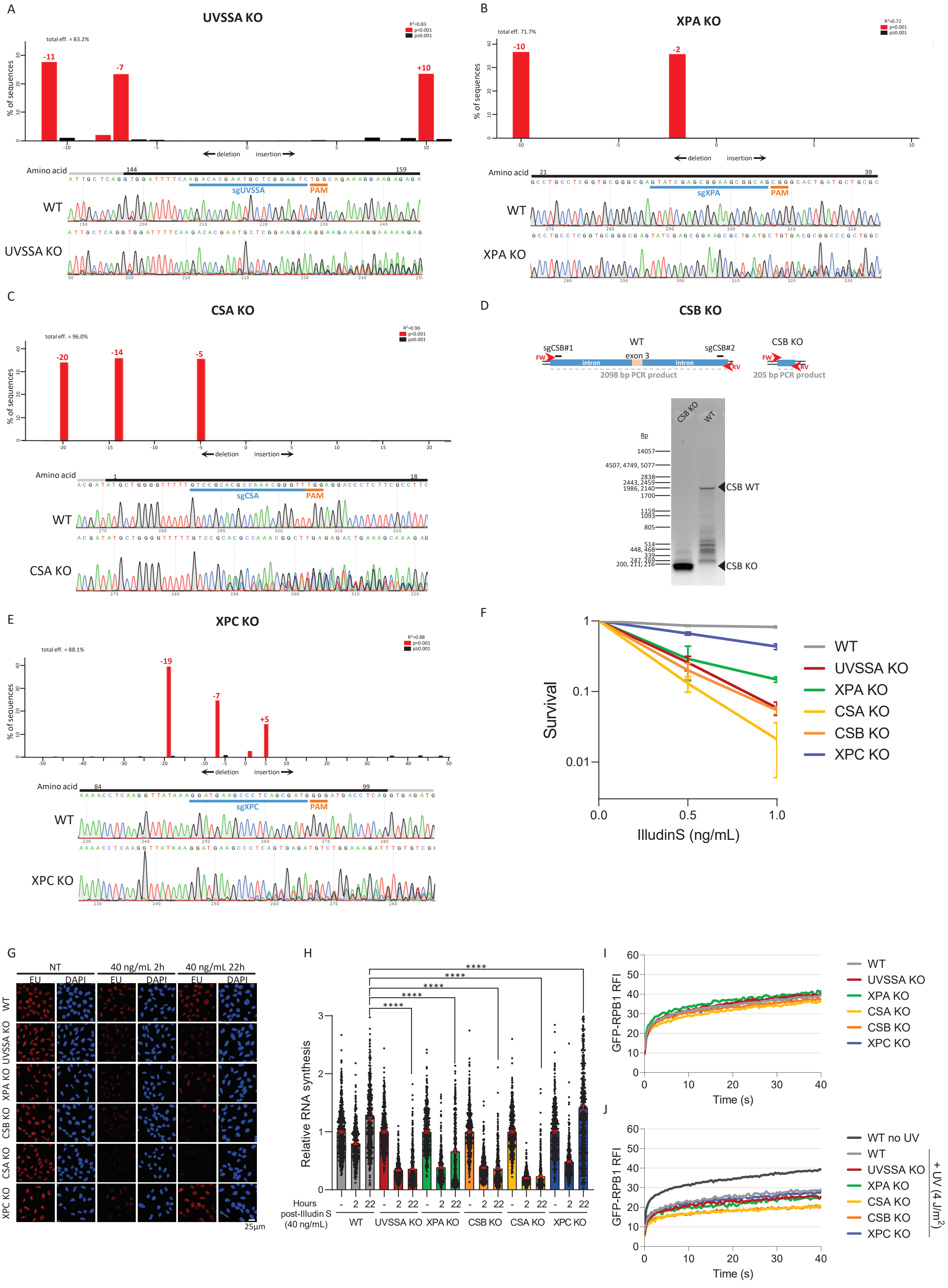

Supplementary Figure S1

A. Genotyping by sequencing results of MRC5$^{GFP\text{-}RPB1}$ UVSSA KO cells used in this study. Position of the used gRNA is indicated by the blue line and PAM sequence is marked with the orange line. Amino acid positions are indicated and intronic and exonic DNA sequences are marked in grey and black, respectively. Observed insertions and deletions in the UVSSA KO cells are marked in red and confirmed by TIDE analysis (upper panel).

B. Genotyping results of MRC5$^{GFP\text{-}RPB1}$ XPA KO cells used in this study as in (A).

C. Genotyping results of MRC5$^{GFP\text{-}RPB1}$ CSA KO cells used in this study as in (A).

D. Genotyping results of MRC5$^{GFP\text{-}RPB1}$ CSB KO cells used in this study. CSB was knocked-out by deleting exon 3 and adjoining introns using two different sgRNAs. CSB KO was assessed by PCR. Position of primers for genotyping is indicated in the cartoon.

E. Genotyping results of MRC5$^{GFP\text{-}RPB1}$ XPC KO cells used in this study as in (A).

F. Relative clonogenic survival of MRC5$^{GFP\text{-}RPB1}$ WT or specified KO cells exposed to the indicated doses of Illudin S (ng/mL). ± SEM, n = 3.

G. Representative images of transcription levels as determined by relative EU incorporation in mock- or Illudin S-treated cells (40 ng/mL). Cells were treated with Illudin S for 3 h. Illudin S was washed away with PBS and transcription was assessed at the indicated times after Illudin S treatment. NT = Non-treated

H. Quantification of transcription restart after Illudin S-induced damage as determined by relative EU incorporation in MRC5$^{GFP\text{-}RPB1}$ WT and indicated KO cells at the specified time points after Illudin S exposure (40 ng/mL) as shown in (G). The relative integrated fluorescence intensity was normalized to mock-treated levels and set to 1. Columns indicate average relative integrated fluorescence intensity and error bars ± SEM. n ≥ 395 cells per condition from 3 independent experiments. ****P< 0.0001.

I. FRAP of GFP-RPB1 seen in (Fig. 1F) zoomed in on the first 40 seconds of the experiment.

J. FRAP of GFP-RPB1 seen in (Fig. 1G) zoomed in on the first 40 seconds of the experiment.

Supplementary Figure S2

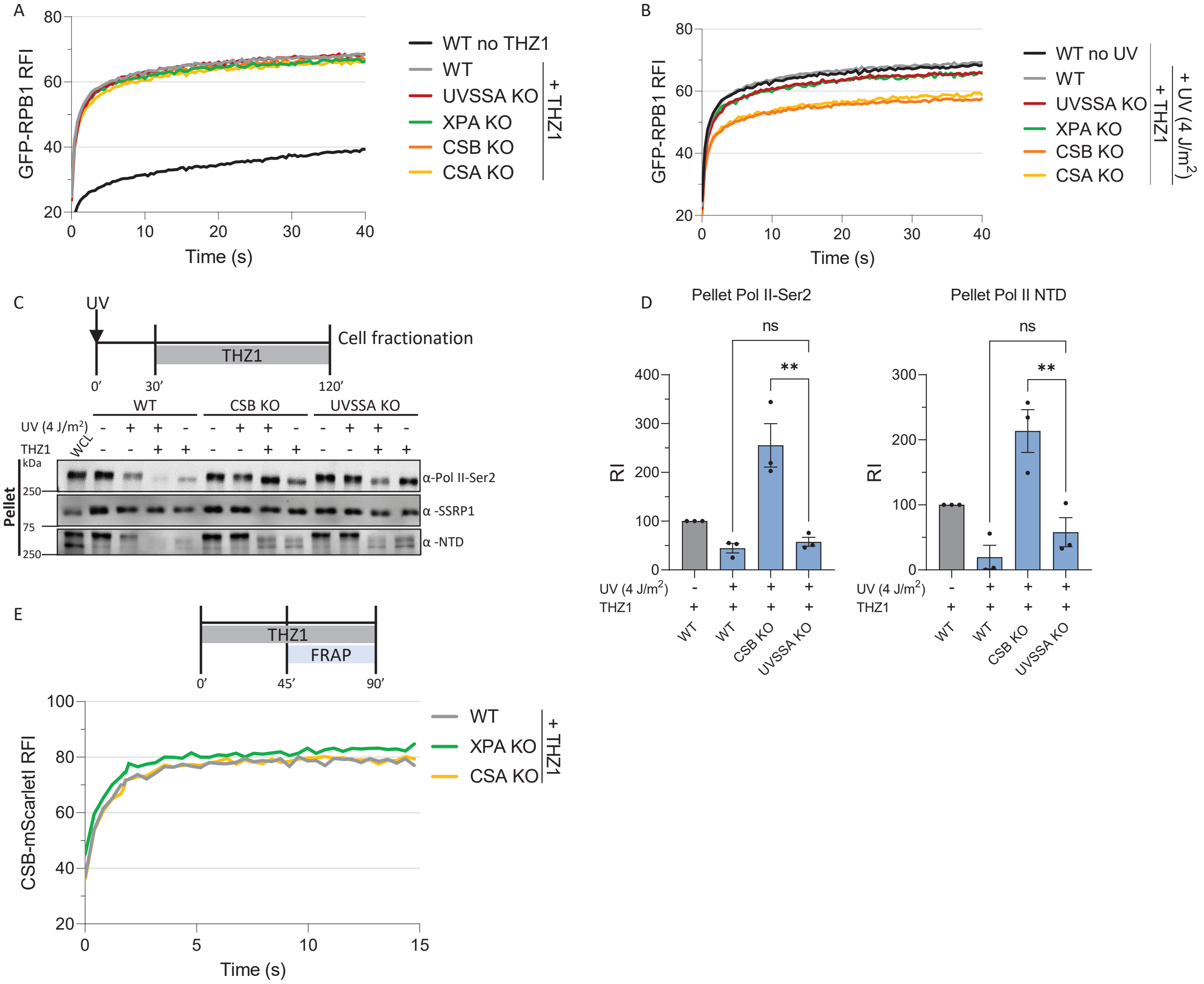

Supplementary Figure S2

A. FRAP of GFP-RPB1 seen in (Fig. 2B) zoomed in on the first 40 seconds of the experiment.

B. FRAP of GFP-RPB1 seen in (Fig. 2C) zoomed in on the first 40 seconds of the experiment.

C. Representative western blot of pellet fraction after cell fractionation in $MRC5^{GFP\text{-}RPB1}$ WT and indicated TC-NER KO cells. NTD and CTD-Ser2-phosphorylated RPB1 (Pol II-Ser2) staining as indicated in chromatin-bound fraction under unperturbed and UV-irradiated (4 J/m^2) conditions with and without THZ1 treatment as indicated to quantify chromatin-bound Pol II. SSRP1 is used as loading control.

D. Quantification of NTD and Pol II-Ser2 signal from 3 independent experiments as in (C). NTD and Pol II-Ser2 signals were normalized to SSRP1 (loading control) signal and to the mock-treated sample for each cell line and set to 100. RI = Relative intensity, ns = not significant, **P<0.01, ± SEM, n = 3.

E. FRAP of CSB-mScarletI in $HCT116^{CSB\text{-}mScarletI}$ WT and TC-NER KO cells, in unperturbed THZ1-treated cells. CSB-mScarletI was bleached in a strip across the nucleus and fluorescence intensity was measured every 0.4 sec for 16 sec. n ≥ 24 cells per condition from at least 2 independent experiments.

Supplementary Figure S3

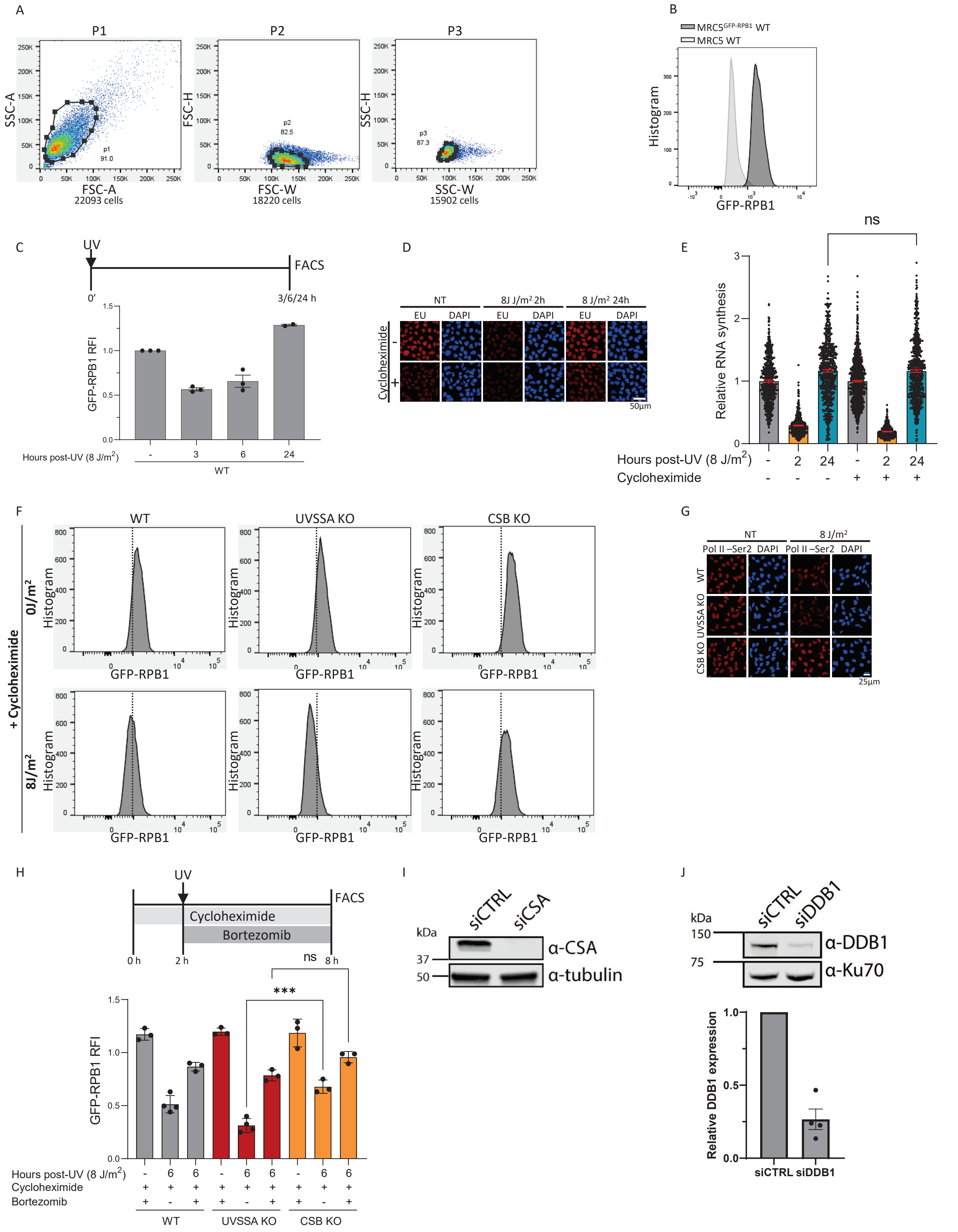

Supplementary Figure S3

A. Representative flow cytometry results showing gating for the analysis of GFP-RPB1 levels. Cell debris was excluded on basis of cell granularity (SSC-A) and size (FSC-A). Exclusion of doublet cells was achieved on basis of plotting FSC-H vs. FSC-W and SSC-H vs. SSC-W.

B. Representative histogram of GFP signal (BB 530_30-A) indicating GFP-RPB1 levels in $MRC5^{GFP-RPB1}$ WT and MRC5 WT (used as negative control) after gating as in (A).

C. Pol II levels in $MRC5^{GFP-RPB1}$ WT cells determined by flow cytometry 3 h, 6 h and 24 h after UV-irradiation (8 J/m^2). GFP-RPB1 levels in UV-treated samples were normalized to mock-treated level which was set to 1. Columns represent average RFI of n ≥ 2 independent experiments ± SEM.

D. Representative images of transcription levels upon 8 h treatment with cycloheximide (100 μM) determined by relative EU incorporation in non-irradiated cells or 2 h and 24 h after UV exposure (8 J/m^2). NT = Non-treated.

E. Quantification of transcription restart after UV damage determined by relative EU incorporation in $MRC5^{GFP-RPB1}$ WT cells at the specified time points after UV exposure (8 J/m^2) as shown in (D). The relative integrated fluorescence intensity of each condition was normalized to mock-treated levels and set to 1. Columns indicate average relative integrated fluorescence intensity and error bars ± SEM. n ≥ 547 cells per condition from at least 3 independent experiments. ns = not significant.

F. Representative histograms of GFP-RPB1 levels after doublet exclusion in $MRC5^{GFP-RPB1}$ WT and indicated TC-NER KO cells. Pol II levels were measured 6 h after mock treatment or UV-irradiation (8 J/m^2). Cycloheximide was added 2 h prior to UV-irradiation and sustained for the duration of the experiment.

G. Representative results of Pol II-Ser2 levels 6 h after UV exposure (8 J/m^2) in $MRC5^{GFPRPB1}$ WT or the indicated TC-NER KO cells. Cells were treated as in (F). NT = Non-treated.

H. Pol II levels in the indicated $MRC5^{GFP-RPB1}$ cells 6 h after UV-irradiation (8 J/m^2) in the presence of cycloheximide (100 μM) and the proteasome inhibitor Bortezomib (5 μM). Cycloheximide was added 2 h prior to UV-irradiation, Bortezomib was added at the time of UV-irradiation and both compounds were maintained for the duration of the experiment. UV-treated samples were normalized to mock-treated samples for each condition which was set to 1. Columns represent average RFI of n ≥ 3 independent experiments ± SEM.

I. Western blot analysis. $MRC5^{GFP-RPB1}$ UVSSA KO cells were stained with CSA antibody in cells transfected with siCSA or siCTRL, which was used as a non-targeting siRNA control. Tubulin was used as a loading control.

J. Upper panel, Western blot analysis. $MRC5^{GFP-RPB1}$ UVSSA KO cells were stained with the indicated antibodies showing DDB1-depletion with siDDB1. siCTRL was used as a non-targeting siRNA control. Ku70 was used as a loading control. Lower panel, Quantification of DDB1 signal from 4 independent experiments. DDB1 signal was normalized to Ku70 (loading control) signal and set to 1.

Supplementary Figure S4

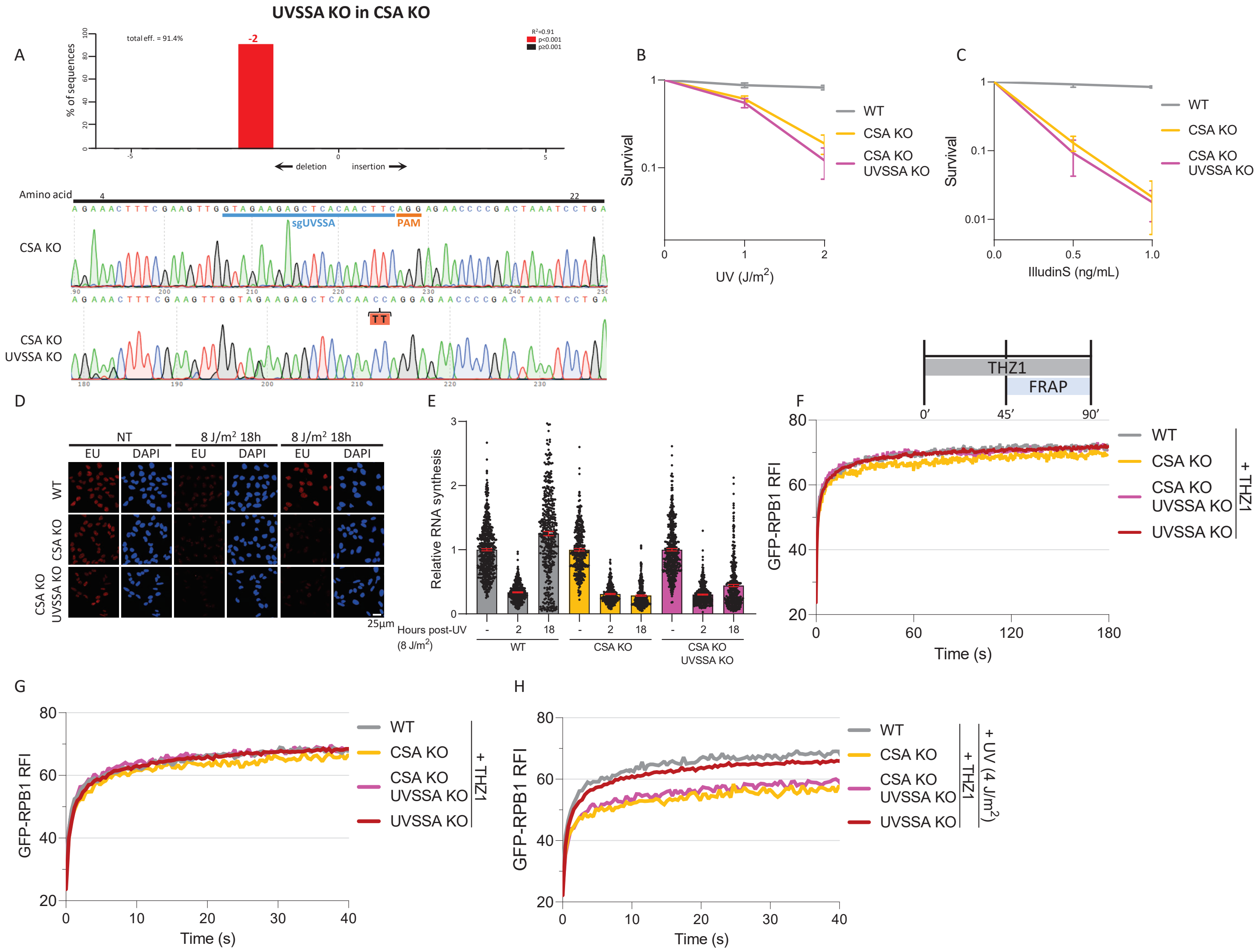

Supplementary Figure S4

A. Genotyping results by sequencing of the UVSSA KO in MRC5$^{GFP-RPB1}$ CSA KO cells. Position of the used gRNA is indicated by the blue line and PAM sequence is marked with the orange line. Observed deletions in the CSA KO/UVSSA KO cells are marked in red and confirmed by TIDE analysis (upper panel).

B. Relative clonogenic survival of MRC5$^{GFP-RPB1}$ WT or specified KO cells exposed to the indicated doses of UV (J/m^2). ± SEM, n ≥ 3.

C. Relative clonogenic survival of MRC5$^{GFP-RPB1}$ WT or specified KO cells exposed to the indicated doses of Illudin S (ng/mL). ± SEM, n ≥ 3.

D. Representative images of transcription levels as determined by relative EU incorporation at the indicated times after UV treatment (8 J/m^2).

E. Quantification of transcription restart after UV-induced damage as determined by relative EU incorporation in MRC5$^{GFP-RPB1}$ WT and indicated KO cells at the specified time points after UV exposure (8 J/m^2) as shown in (D). The relative integrated fluorescence intensity was normalized to mock-treated levels and set to 1. Columns indicate average relative integrated fluorescence intensity and error bars ± SEM. n ≥ 300 cells per condition from 3 independent experiments.

F. FRAP analysis of GFP-RPB1 of the indicated cell lines after THZ1 treatment as indicated in the cartoon. GFP-RPB1 was bleached and fluorescence intensity was measured every 0.4 sec for 3 min, background-corrected and normalized to pre-bleach fluorescence intensity, which was set to 100. GFP-RPB1 mobility of unperturbed UVSSA KO cells from (Fig. 2B) cells is plotted in red for reference. Average Relative Fluorescence Intensity (RFI) of n ≥ 31 cells per condition from 3 independent experiments.

G. FRAP of GFP-RPB1 shown in (F) zoomed in on the first 40 seconds of the experiment.

H. FRAP of GFP-RPB1 seen in (Fig. 3H) zoomed in on the first 40 seconds of the experiment.

Supplementary Figure S5

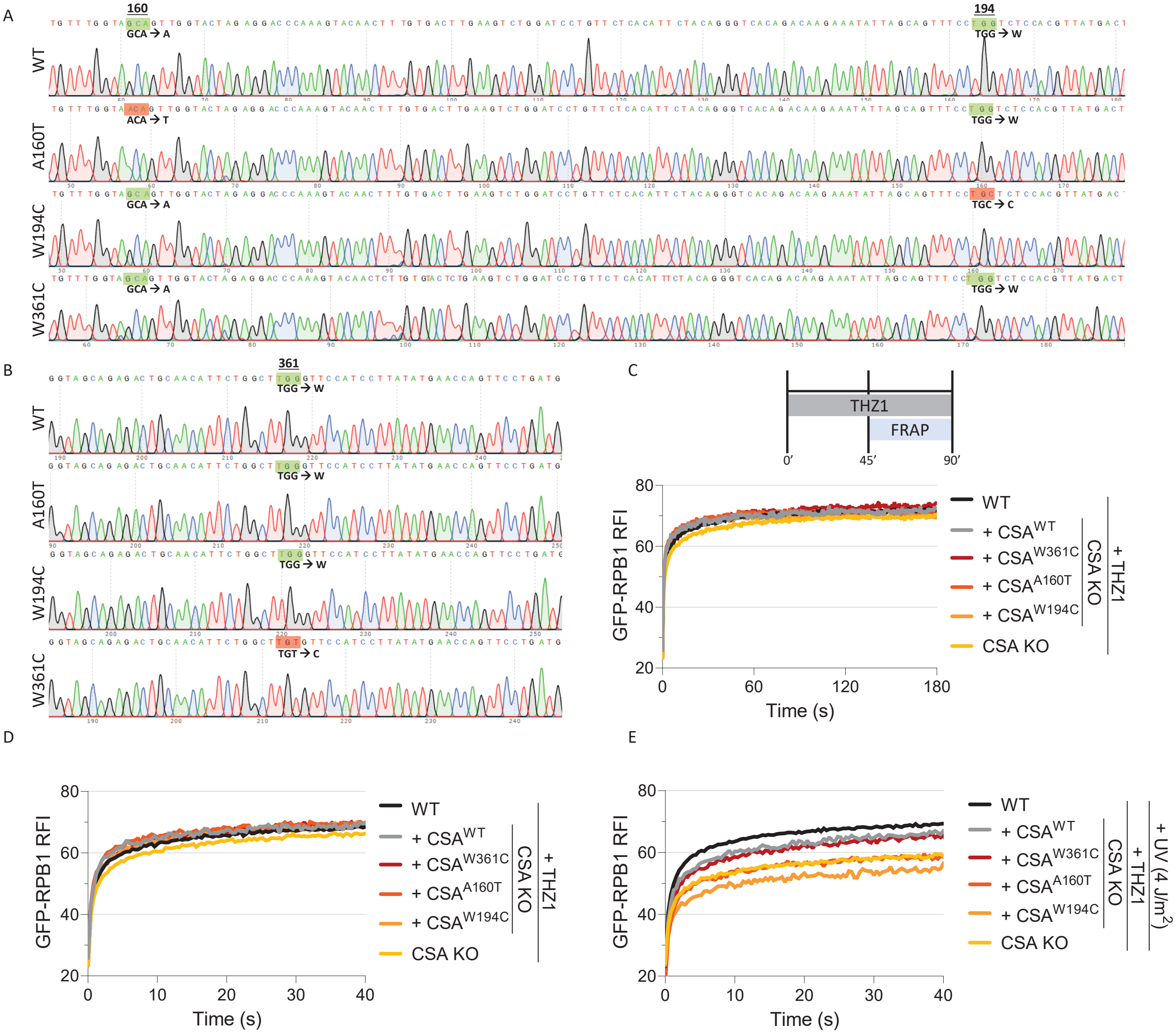

Supplementary Figure S5

A. Genotyping results by sequencing of MRC5$^{GFP\text{-}RPB1}$ CSA KO cells complemented with WT CSA constructs or the CSA harboring the point mutations A160T, W194C or W361C. The observed A160T and W194C point mutations are marked in red. The primers for genotyping were designed over exon-exon boundaries to ensure PCR of the transgene.

B. Genotyping as in (A), but showing the W361C point mutation.

C. FRAP of GFP-RPB1 upon CDK7 inhibition with THZ1 of the different CSA point mutants expressed in CSA KO cells. GFP-RPB1 mobility of unperturbed WT and CSA KO cells is plotted in black and yellow, respectively, for comparison. Average RFI of n ≥ 16 cells per condition from at least 2 independent experiments.

D. FRAP of GFP-RPB1 shown in (C) zoomed in on the first 40 seconds of the experiment.

E. FRAP of GFP-RPB1 seen in (Fig. 4C) zoomed in on the first 40 seconds of the experiment.

Supplementary Figure S6

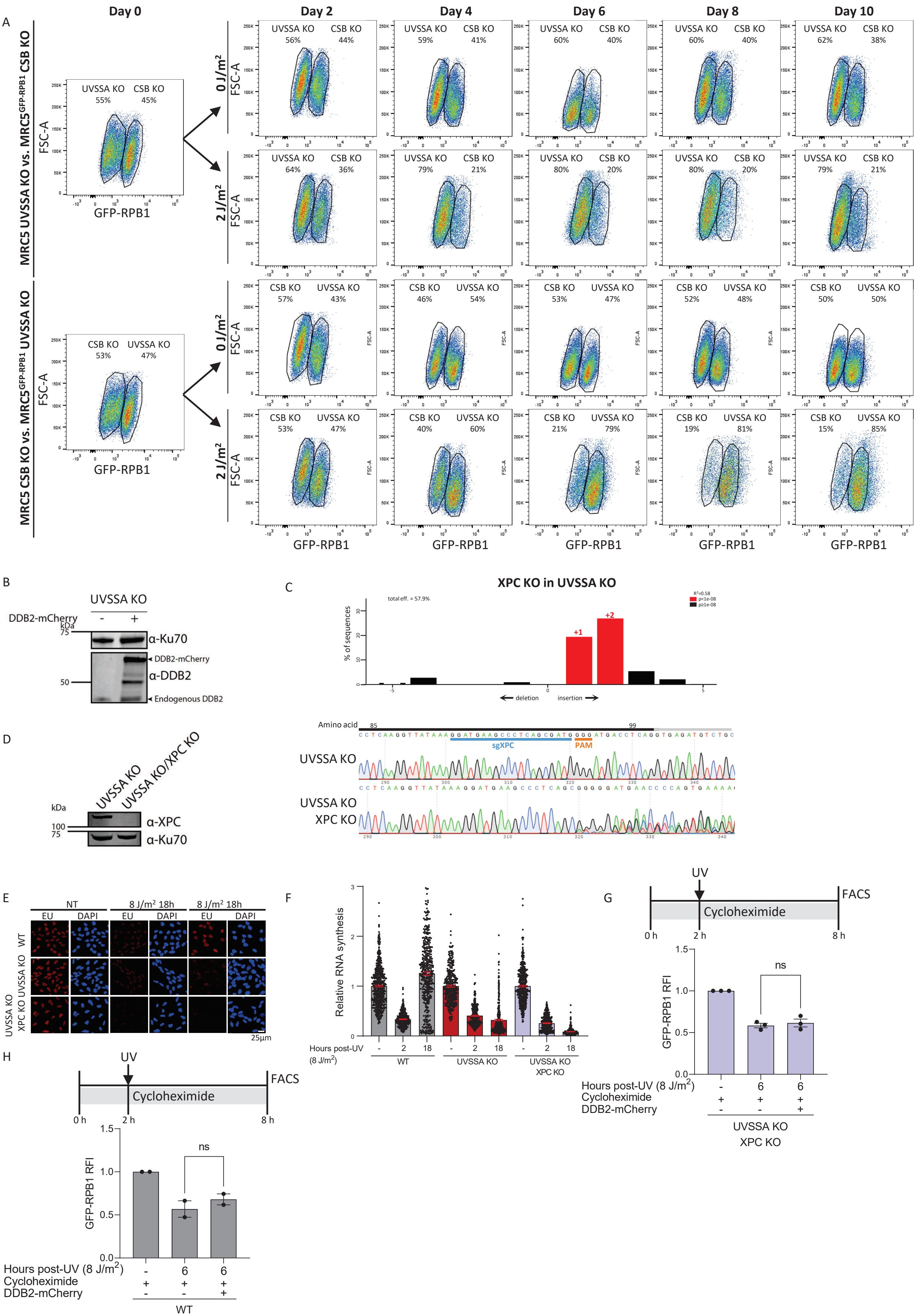

Supplementary Figure S6

A. Representative results of flow cytometry gating of competitive growth assay of MRC5 UVSSA KO with MRC5$^{GFP\text{-}RPB1}$ CSB KO cells (top panel) or MRC5$^{GFP\text{-}RPB1}$ UVSSA KO with MRC5 CSB KO cells. As indicated, cells remained untreated or were UV-irradiated for 10 successive days with 2 J/m^2 UV. Cells were analyzed by flow cytometry at the indicated days. Relative ratios between GFP-RPB1-expressing and not expressing cell populations are indicated.

B. Western blot analysis. MRC5$^{GFP\text{-}RPB1}$ UVSSA KO cells were stained with DDB2 antibody to show expression of DDB2-mCherry. Ku70 was used as a loading control.

C. Genotyping results by sequencing of MRC5$^{GFP\text{-}RPB1}$ XPC KO generated in UVSSA KO cells. Position of the used gRNA is indicated by the blue line and PAM sequence is indicated by the orange line. Observed insertions are marked in red and determined by TIDE analysis (upper panel).

D. Western blot analysis. MRC5$^{GFP\text{-}RPB1}$ UVSSA KO and UVSSA/XPC double KO cells were stained with XPC antibody. Ku70 was used as a loading control.

E. Representative images of transcription levels as determined by relative EU incorporation at the indicated times after UV treatment (8 J/m^2).

F. Quantification of transcription restart after UV-induced damage as determined by relative EU incorporation in MRC5$^{GFP\text{-}RPB1}$ WT and indicated KO cells at the specified time points after UV exposure (8 J/m^2) as shown in (E). The relative integrated fluorescence intensity was normalized to mock-treated levels and set to 1. Columns indicate average relative integrated fluorescence intensity and error bars ± SEM. n ≥ 321 cells per condition from 3 independent experiments.

G. Pol II levels in MRC5$^{GFP\text{-}RPB1}$ UVSSA/XPC double KO cells 6 h after UV-irradiation (8 J/m^2) in the presence of cycloheximide (100 μM), added 2 h prior to UV-irradiation and maintained for the duration of the experiment. DDB2 was transiently overexpressed in these cells. UV-treated samples were normalized to mock-treated samples for each condition which was set to 1. Columns represent average RFI of n = 3 independent experiments ± SEM. ns = not significant.

H. Pol II levels in MRC5$^{GFP\text{-}RPB1}$ WT cells. DDB2 was transiently overexpressed in these cells which were treated as in (G). Columns represent average RFI of n = 2 independent experiments ± SEM. ns = not significant.

Supplementary table S1. sgRNA sequences used to generate KO cell lines

Generated Cell line	sgRNA	sgRNA Sequence	First mention
MRC5 $^{GFP\text{-}RPB1}$ WT	N.A	N.A	(1)
MRC5 $^{GFP\text{-}RPB1}$ CSA KO	CSA	GTCCGCACGCCAAACGGGTT	(2)
MRC5 $^{GFP\text{-}RPB1}$ CSB KO	CSB	#1: GCGAGGGCTGAACGGGATGG<br>#2: GCTTTGGAAAACTTAAGGGT<br>The two sgRNAs were combined to remove complete exon 3	(2)
MRC5 $^{GFP\text{-}RPB1}$ UVSSA KO	UVSSA	AGACACGAATGCTCGGAGTC	(3)
MRC5 $^{GFP\text{-}RPB1}$ XPA KO	XPA	GTATCGAGCGGAAGCGGCAG	(2)
MRC5 $^{GFP\text{-}RPB1}$ XPC KO	XPC	GGATGAAGCCCTCAGCGATG	(2)
MRC5 $^{GFP\text{-}RPB1}$ CSA/UVSSA KO	UVSSA	GTAGAAGAGCTCACAACTTC	This paper
MRC5 $^{GFP\text{-}RPB1}$ UVSSA/XPC KO	XPC	GGATGAAGCCCTCAGCGATG	This paper
HCT116 $^{CSB\text{-}mScarletI}$ WT	N.A	N.A	(4)
HCT116 $^{CSB\text{-}mScarletI}$ CSA KO	CSA	GTCCGCACGCCAAACGGGTT	(5)
HCT116 $^{CSB\text{-}mScarletI}$ XPA KO	XPA	GTATCGAGCGGAAGCGGCAG	(5)

1. B. Steurer, *et al.*, Live-cell analysis of endogenous GFP-RPB1 uncovers rapid turnover of initiating and promoter-paused RNA Polymerase II. *PNAS* **115**, E4368–E4376 (2018).

2. B. Steurer, *et al.*, DNA damage-induced transcription stress triggers the genome-wide degradation of promoter-bound Pol II. *Nat. Commun.* **13** (2022).

3. M. van Sluis, *et al.*, Transcription-coupled DNA–protein crosslink repair by CSB and CRL4CSA-mediated degradation. *Nat. Cell Biol.* **26** (2024).

4. M. E. Geijer, *et al.*, Elongation factor ELOF1 drives transcription-coupled repair and prevents genome instability. *Nat. Cell Biol.* **23**, 608–619 (2021).

5. D. Zhou, Q. Yu, R. C. Janssens, J. A. Marteijn, Live-cell imaging of endogenous CSB-mScarletI as a sensitive marker for DNA-damage-induced transcription stress. *Cell Reports Methods* **4**, 100674 (2024).

Supplementary table S2. Primers for genotyping

Cell line	Primer	Sequence
MRC5 $^{GFP\text{-}RPB1}$ CSA KO and<br>HCT116 $^{CSB\text{-}mScarletI}$ CSA KO	CSA_1 FW<br>CSA_1 RV	CTTGACAGCGATCTCCACAAG<br>CAAGCCACACAGCATTAGAG
MRC5 $^{GFP\text{-}RPB1}$ CSA KO + CSA^{A160T} and<br>MRC5 $^{GFP\text{-}RPB1}$ CSA KO + CSA^{W194C}	CSA_2 FW<br>CSA_2 RV	ATACATTACAAACTGCGGAT<br>GCAGTGTTTGCTGATTCAAC
MRC5 $^{GFP\text{-}RPB1}$ CSA KO + CSA^{W361C}	CSA_3 FW<br>CSA_3 RV	CACACTTGTGAACTATGGAA<br>CACCCTTTCCTTCTTCATCA
MRC5 $^{GFP\text{-}RPB1}$ CSB KO	CSB FW<br>CSB RV	GGCAGTGTCAGGTAAGCAAG<br>TCAGTTCTGCCATCCCAACT
MRC5 $^{GFP\text{-}RPB1}$ UVSSA KO	$UVSSA_1$ FW<br>$UVSSA_1$ RV	CATTCTCCTGCCTCAATCTC<br>CCTGTGCCTGGCATCTCTG
MRC5 $^{GFP\text{-}RPB1}$ CSA/UVSSA KO	$UVSSA_2$ FW<br>$UVSSA_2$ RV	TAAAACTGCAATCACCGCCG<br>ACCCCAGAACATGACCACC
MRC5 $^{GFP\text{-}RPB1}$ XPA KO and<br>HCT116 $^{CSB\text{-}mScarletI}$ XPA KO	XPA FW<br>XPA RV	CCTTCTCCCGGATGACAAGA<br>CGAGGTGCAGCGAAGAAG
MRC5 $^{GFP\text{-}RPB1}$ XPC KO	XPC_1 FW<br>XPC_1 RV	TGGAGATGGGGTTTGGAGAC<br>AGTAGAAAGAGCACTGGACCA
MRC5 $^{GFP\text{-}RPB1}$ UVSSA/XPC KO	XPC_2 FW<br>XPC_2 RV	GAAGCTGAGAGCTGGTTGCT<br>ACCTGGGTTCTCCTGCTTTT

Supplementary table S3. siRNA sequences

**siRNA**	**Sequence**
CSA	CAGACAAUCUUAUUACACA
DDB1	UGAUAAUGGUGUUGUGUUU
Elongin A	GCACAGAACCCAGAGAAA
NEDD4	AUGGAGUUGAUUAGAUUACAA
Non-targeting control	UGGUUUACAUGUCGACUAA

Supplementary table S4. Primary antibody list and working solutions

Antibody	Host	Source	WB Dilution	IF Dilution
CSA/ERCC8	Rb	Abcam, ab240096	1:1000	N.A
CSB/ERCC6	Rb	Aboline, ABIN2855858	1:1000	N.A
DDB1	Rb	Novus Bio, NBP2-75465	1:1000	N.A
DDB2	m	Abcam, ab181136	1:1000	N.A
FLAG	Rb	Sigma Aldrich, F7425	1:1000	N.A
Ku70	Rb	Santa Cruz, sc-17789	1:1000	N.A
RPB1-NTD	Rb	Cell Signaling, D8L4Y	1:1000	N.A
RPB1-pSer2	Rb	Abcam, ab5095	1:1000	1:1000
SSRP1	m	Biolegend, 609702	1:5000	N.A
Tubulin	m	Sigma Aldrich, B512	1:10000	N.A
XPA	Rb	Genetex, GTX103168	1:1000	N.A
XPC	Rb	Bethyl Laboratories, A301-122A	1:2000	N.A

Supplementary table S5. Secondary antibody list and working solutions

Source, Reference	Antibody	WB Dilution	IF Dilution
Sigma, Sab4600215	Anti-rabbit, CF IRDye 770	1:10000	N.A
Sigma, Sab4600199	Anti-mouse, CF IRDye 680	1:10000	N.A
Invitrogen, A11012	Anti-rabbit, Alexa Fluor 594	N.A	1:1000
